# Cytoplasmic Relaxation of Active Eph Controls Ephrin Shedding by ADAM10

**DOI:** 10.1371/journal.pbio.1000215

**Published:** 2009-10-13

**Authors:** Peter W. Janes, Sabine H. Wimmer-Kleikamp, Achilleas S. Frangakis, Kane Treble, Bettina Griesshaber, Ola Sabet, Markus Grabenbauer, Alice Y. Ting, Paul Saftig, Philippe I. Bastiaens, Martin Lackmann

**Affiliations:** 1 Department of Biochemistry and Molecular Biology, Monash University, Victoria, Australia; 2 European Molecular Biology Laboratory, Heidelberg, Germany; 3 Department of Systemic Cell Biology, Max Planck Institute of Molecular Physiology, Dortmund, Germany; 4 Department of Chemistry, Massachusetts Institute of Technology, Cambridge, Massachusetts, United States of America; 5 Biochemical Institute, Christian-Albrecht-University, Kiel, Germany; Salk Institute for Biological Studies, United States of America

## Abstract

Release of cell surface-bound ligands by A-Disintegrin-And-Metalloprotease (ADAM) transmembrane metalloproteases is essential for signalling by cytokine, cell adhesion, and tyrosine kinase receptors. For Eph receptor ligands, it provides the switch between cell-cell adhesion and repulsion. Ligand shedding is tightly controlled by intrinsic tyrosine kinase activity, which for Eph receptors relies on the release of an inhibitory interaction of the cytoplasmic juxtamembrane segment with the kinase domain. However, a mechanism linking kinase and sheddase activities had remained elusive. We demonstrate that it is a membrane-proximal localisation of the latent kinase domain that prevents ephrin ligand shedding in *trans*. Fluorescence lifetime imaging microscopy and electron tomography reveal that activation extends the Eph receptor tyrosine kinase intracellular domain away from the cell membrane into a conformation that facilitates productive association with ADAM10. Accordingly, EphA3 mutants with constitutively-released kinase domains efficiently support shedding, even when their kinase is disabled. Our data suggest that this phosphorylation-activated conformational switch of EphA3 directly controls ADAM-mediated shedding.

## Abstract 2

Novel imaging strategies reveal a conformational shift in a receptor tyrosine kinase domain that controls ligand shedding by an ADAM metalloprotease.

## Author Summary

The Eph transmembrane receptors are part of the receptor tyrosine kinase family and play important roles in communication between neighbouring cells. An Eph receptor binds to its ligand, membrane-tethered ephrin, on a neighbouring cell so as to form a stable complex and activate downstream signalling events. One such event is regulation of ADAM10, a transmembrane protease of the ADAM metalloprotease family, which provides a feedback mechanism to Eph signalling. ADAM10 is located on Eph-expressing cells and cleaves ephrin from its membrane tether on the opposite cell (through its so-called sheddase activity), thereby separating the cell-cell connection and allowing the signalling complex to internalise. In other biological contexts, activity of the ADAM metalloprotease family underlies signalling mechanisms such as oncogenic EGF-receptor transactivation, adhesion molecule shedding and cytokine/chemokine release. In general, ADAM function is enhanced when receptor tyrosine signalling is active and repressed when tyrosine kinase signalling is inhibited. However, the mechanism through which receptor tyrosine kinase signalling regulates ADAM10, have remained elusive. By combining fluorescence lifetime imaging microscopy (FLIM) and electron microscopic tomography of EphA3, we have demonstrated in live cells at molecular resolution that tyrosine phosphorylation of activated EphA3 triggers a measurable movement of the kinase domain away from the plasma membrane. Only this conformation of the EphA3 kinase domain away from the plasma membrane permits ADAM10 to come close enough to EphA3 so that it can reach its tightly EphA3-bound substrate, ephrin-A5. Our findings delineate a new regulatory concept in cell-cell communication, whereby control over proteolytic sheddase activity is provided by an activation-induced switch in the conformation of the cytoplasmic domain of a receptor tyrosine kinase, rather than by a cytosolic signalling pathway.

## Introduction

The ADAM (A-Disintegrin-And-Metalloprotease) transmembrane proteases fulfil essential functions during normal and pathological tissue- and organ-development [1]. ADAM10 and 17 in particular are widely expressed and knock-out mice lacking expression of either gene show severe, lethal defects in early development, in the formation of somites and the central nervous system (ADAM10) and the cardiovascular system (ADAM10/17). They have important roles in receptor tyrosine kinase (RTK) and Notch signalling, highlighted by phenotypic resemblance of ADAM 10/17 knockouts with those of Notch, the epidermal growth factor receptor (EGFR) and EGFR ligands [2],[3],[4],[5]. ADAM10 and 17 both target a range of EGFR ligands with distinct specificities [6], while ADAM10 cleaves both Notch and its ligand delta, as well as other targets with prominent roles in disease including amyloid precursor protein, interleukin 6 receptor [5], cadherins [7], and ligands for Eph RTKs (Ephs) [8],[9].

Ephs and their membrane bound ligands (ephrins) control cell positioning during normal and oncogenic development by modulating cell-cell adhesion and cell-cell segregation or repulsion [10]. Similar to ADAMs, they function in developmental processes including somite formation, neural and cardiovascular development [11],[12], which, together with their common expression patterns, supports functional evidence for the critical role of ADAM10 in Eph biology [8],[9]. Eph function relies on the direct contact between Eph- and ephrin-expressing cells, which triggers the assembly of signalling clusters at the cell-cell interface [13] and initiates Eph “forward” and ephrin “reversed” signals into the respective cells [10]. For repulsion to proceed it is essential that the multivalent [14],[15] signalling complexes that tether Eph- and ephrin-expressing cells are disrupted, allowing the cells to retract via ensuing actin cytoskeletal rearrangement [16]. In the case of EphA/ephrin-A signalling clusters it was demonstrated that ephrin-shedding by ADAM10, constitutively associated with Ephs on the opposing cell [9], is required for repulsion to occur [8],[9].

ADAM proteins are produced as inactive precursors that become catalytically active upon removal of the prodomain during secretion. Interestingly, there is little evidence for a substrate cleavage sequence motif, and the regulation of proteolytic specificity is achieved by interaction of substrates with the non-catalytic disintegrin and cysteine-rich extracellular ADAM domains. For ephrin cleavage, a substrate recognition module within the cysteine-rich domain of ADAM10 specifically binds only the high-affinity ligand-receptor complex to ensure that only Eph receptor-bound ephrins are cleaved [9].

n addition to this direct control of ADAM10-facilitated shedding, substantial evidence documents intracellular regulation of ADAM proteases [4],[5]. ADAM activity is enhanced upon activation of (receptor) tyrosine kinase signalling by growth factors, phorbol esters, or phosphatase inhibitors, while tyrosine kinase inhibitors or dominant-negative RTK mutants attenuate ADAM activity [4]. This protein kinase-controlled ADAM activity is an essential component of the autocrine, mitogenic signalling that is triggered by G-protein coupled [17],[18] or stress-induced EGF receptor transactivation [17],[19]. Conversely, ADAM shedding of L-selectin during leukocyte trafficking [20] is blocked by Calmodulin (CaM), via its binding to the 17-residue L-selectin intracellular domain (ICD), while CaM inhibitors trigger shedding of the L-selectin ectodomain [21]. Surprisingly, while ADAM family members harbour potential protein docking motifs [4], cytoplasmic-truncated ADAM17 is fully functional [22], and signalling mechanisms regulating ADAM activity have remained elusive.

To elaborate the intracellular regulation of ADAMs by RTK signalling we investigated ADAM10 catalysed ephrin-A5 cleavage that is mediated by EphA3-expressing cells, as previous studies demonstrated that this sheddase activity depends on ephrins binding and activating the EphA3 RTK [8],[9]. Our results suggest that a conformational change in the EphA3 ICD, which upon activation moves the kinase domain away from the plasma membrane, relieves a steric hindrance preventing productive association with ADAM10. We demonstrate that this loss of steric hindrance, resulting from extension of the active Eph ICD, rather than classical signalling via intermediate proteins, regulates the sheddase activity of ADAM10.

## Results

### Intracellular Regulation of ADAM10-Mediated Ephrin Cleavage

**Figure 1:**
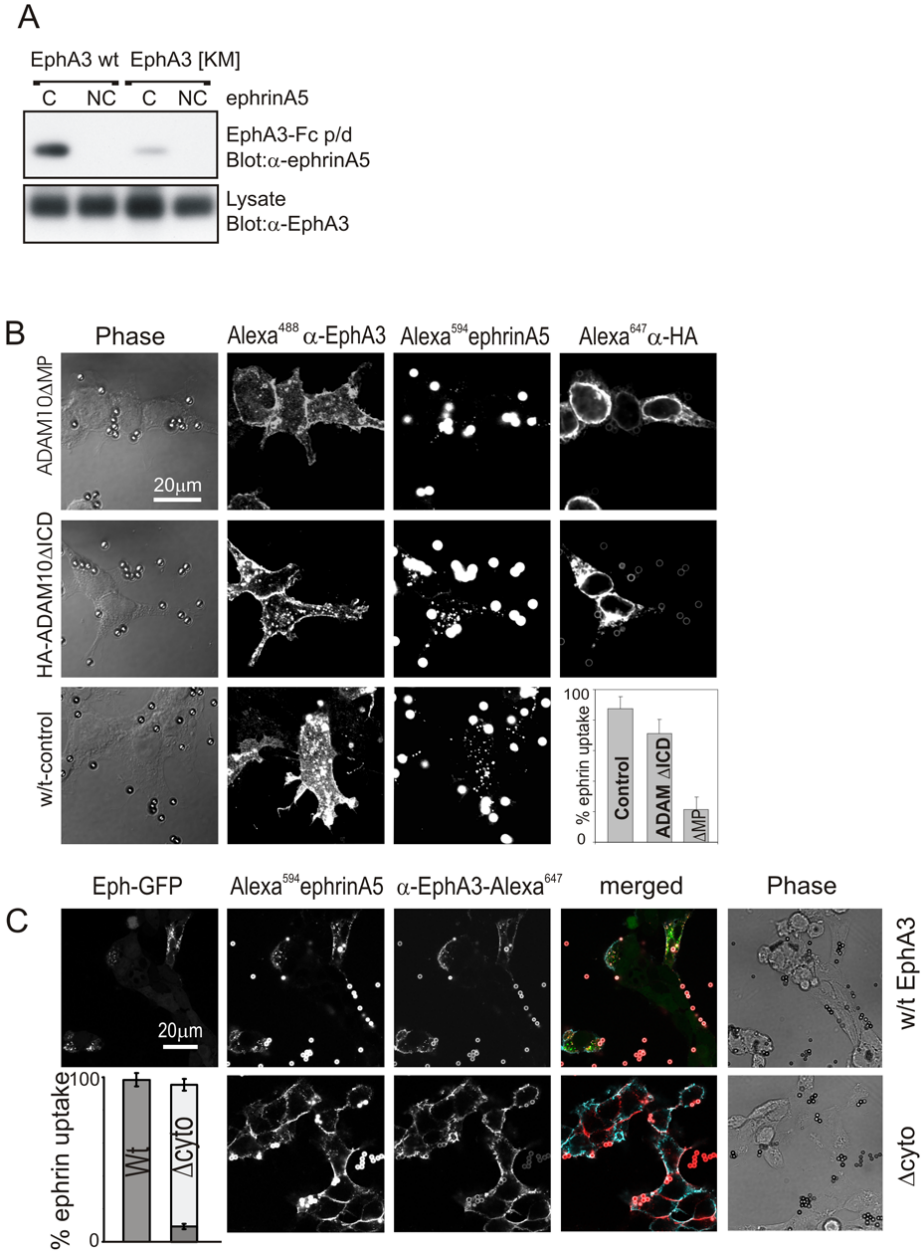
Ephrin shedding is inhibited by lack of kinase activity but not by cytoplasmic truncation of EphA3 or ADAM10. (A) EphA3 kinase activity is required for effective ephrin cleavage. HEK293T cells expressing EphA3 Wt or EphA3[KM] (with a mutated ATP-binding site, K_653_→M) were treated with clustered (C) or non-clustered (NC) ephrin-A5-Fc. Cleaved ephrin-A5 was extracted by EphA3-Fc pulldown and recovered ephrin-A5 and total cell extracts were immunoblotted as indicated. Low level ephrin-A5 shedding observed with ephrin-A5-Fc -treated EphA3[KM] cells is likely due to endogenous EphA3 present in parental HEK293T cells [45]. (B) Truncation of the ADAM10 ICD (ΔICD) does not affect ephrin-A5 cleavage and internalisation. Cells expressing endogenous ADAM10 and EphA3-GFP were transfected with HA-ADAM10[ΔMP] (top), HA-ADAM10[ΔICD] (middle), or control vector (bottom) and exposed to Alexa^594^ephrin-A5-Fc coated beads. EphA3-GFP, internalised ephrin-A5, and HA-ADAM staining (Alexa^647^) were imaged by confocal microscopy. The relative ephrin-A5 labelling of receptor-expressing cells (mean+/−SEM), with or without ADAM10[ΔICD] or ADAM10[ΔMP] co-expression, is shown. (C) Cleavage of ephrin-A5 from conjugated beads in the presence of EphA3[Δcyto]-expressing cells. HEK293T cells expressing Wt EphA3-GFP or EphA3[Δcyto] as indicated were incubated with Alexa^594^ephrin-A5-Fc-conjugated beads before staining with Alexa^647^anti-EphA3 antibody. Confocal microscope images show representative cells stained with cleaved ephrin-A5. Pseudocolours in the merged images illustrate: green, EphA3-GFP; red, Alexa^594^ephrin-A5; blue, Alexa647anti-EphA3 antibody. The graph shows the relative ephrin-labelling of cells (mean+/−SEM), with cells containing internalised ephrin shown in dark grey and cell-surface ephrin in light gray.

Eph activation, phosphorylation, and signalling relies on the assembly of multimeric Eph/ephrin complexes [11],[12]. For in vitro experiments, recombinant proteins comprising two ephrin extracellular domains fused onto the Fc portion of human IgG (ephrin-Fc) can be clustered with anti-Fc antibodies to elicit Eph activation [23],[24],[25]. We tested the cleavage of clustered or non-clustered ephrin-A5-Fc by cell surface ADAM10 in cultures of HEK293T (transformed human embryonic kidney cells) cells expressing either wild type (Wt) EphA3 or mutant, kinase-inactive EphA3[K_653_M]. Immunoblot analysis confirmed that the release of ephrin-A5 from the Fc complexes was dependent on pre-clustering and indeed was greatly reduced in cultures of cells expressing the kinase-inactive EphA3 mutant (Figure 1A, Figure S1A).

Surprisingly, however, co-immunoprecipitation analysis revealed robust binding of ADAM10 to an EphA3 mutant lacking the whole ICD (EphA3[ΔICD], Figure S1B). This implies that the ADAM10/EphA3 association, which is necessary for ephrin cleavage and involves constitutive as well as ephrin-augmented interactions of their extracellular domains [8],[9], does not require the contribution of the EphA3 ICD. We therefore assessed if cytoplasmic-truncated ADAM10[ΔICD] or EphA3[ΔICD] could catalyse cleavage of Alexa-labelled ephrin-A5-Fc that had been conjugated to Protein-A-coated Dynabeads, an experimental approach previously used to characterise ephrin shedding by ADAM10 [9]. In agreement with earlier studies [8], over-expression of non-functional ADAM10 lacking the MP domain (ADAM10ΔMP) acts as a dominant negative protein to effectively prevent ephrin shedding (Figure 1B). By contrast, over-expression of cytoplasmic-truncated ADAM10[ΔICD] did not notably affect ephrin-A5 cleavage and internalisation (Figure 1B), indicating that the ADAM ICD may not be required for its sheddase activity [22].

Likewise, cells over-expressing EphA3[ΔICD] efficiently supported ephrin-A5 shedding from Dynabeads, as evident from the marked cell surface labelling with fluorescent ephrin that had been released from the beads (Figure 1C). The lack of efficient internalisation of the cleaved ephrin-A5 into cells in this case suggested that the EphA3 ICD is required for internalisation of the ligand/receptor complex, but is not essential for ligand cleavage. Cell surface labelling was efficiently blocked by ADAM metalloprotease inhibitors (Figure S2), consistent with ADAM-dependent shedding of ephrin-A5 [9].

### Active EphA3 Conformation rather than Kinase Activity Correlates with ADAM10-Mediated Ephrin Cleavage

To reconcile the observations that ephrin cleavage requires Eph kinase activity but still occurs in the absence of the entire ICD, we considered recent studies demonstrating an activation- and phosphorylation-dependent release of the Eph juxtamembrane (JM) segment from an inhibitory interaction with the kinase domain [26]. The existence of this structural switch, which converts a static/constrained conformation of the JM domain into a dynamic/relaxed one [26],[27], was recently confirmed also for EphA3 [28].

**Figure 2:**
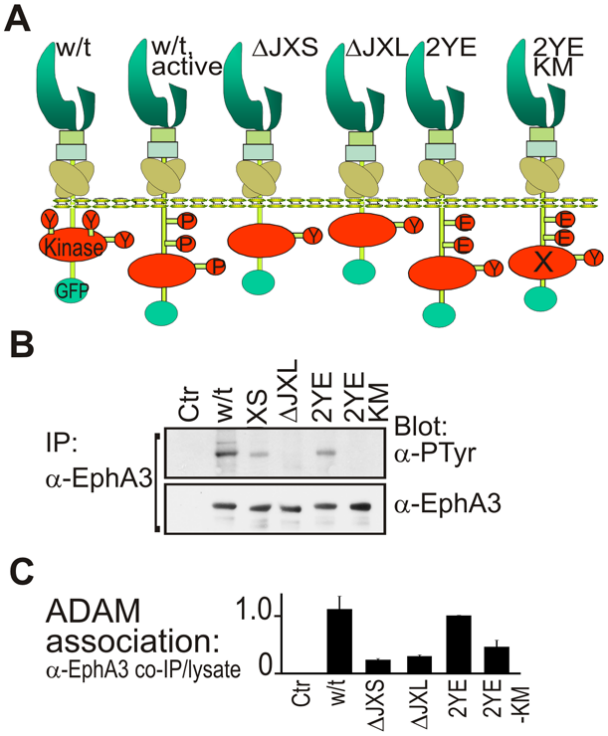
Mutation of the JM domain affects EphA3 phosphorylation and ADAM10 association. (A) Schematic structure of Wt EphA3-GFP and derived ICD mutants (see text for details) that were used in these studies. Y, tyrosine; P, phospho-tyrosine; E, glutamate (pseudophosphorylation) ; X, inactive kinase. (B) Tyrosine phosphorylation of Wt and mutant EphA3. EphA3 immunoprecipitates from lysates of ephrin-A5-Fc-stimulated cells were immunoblotted with anti-phosphotyrosine and anti-EphA3 antibodies as indicated. (C) ADAM10 association with Wt and mutant EphA3. ADAM10 immunoprecipitates and total cell lysates from Wt or mutant (as indicated) EphA3-transfected cells (ephrin-A5-treated) were analysed for EphA3 and ADAM10 by immunoblot (see Figure S4B). The average ratio (+/−SD) of EphA3 in precipitates relative to lysates (*n* = 2 experiments) is plotted, with EphA3[2YE] as internal reference.

We hypothesized that the inactive/constrained JM segment positions the kinase domain close to the membrane, a configuration that imparts a steric obstruction to the productive ADAM10/EphA3 interaction and thereby controls ephrin shedding. To test this hypothesis we examined whether forced approximation of the EphA3 kinase domain to the plasma membrane affects ADAM10 association and function. For these experiments we designed a series of EphA3 mutants (Figure 2A) including: i) “EphA3[ΔJX]” short (“S”), lacking JM residues 591–614 and replicating the previously-reported EphB2[Δ599–621] [26]; ii) “EphA3[ΔJX]” long (“L”) lacking all JM residues 567–614; iii) “EphA3[2YE]” where Y→E substitutions of JM tyrosines generate an unfolded JM domain [26]; and iv) EphA3[2YE-KM], a kinase-inactive form of EphA3[2YE]. All mutants were expressed at the cell surface and functional in ephrin-A5 binding (Figure S3). Loss of the JM tyrosines Y596 and Y602 in the truncated (EphA3[ΔJXS]) or Y→E substituted (EphA3[2YE]) JM domain reduced ephrin-A5 induced phosphorylation (Figure 2B) without loss of EphA3 kinase activity (Figure S4A). By contrast, only marginal phosphorylation of EphA3[ΔJXL] (Figure 2B) suggests that the very close proximity of the kinase domain to the inner membrane leaflet impedes the substrate interaction for this mutant.

Importantly, all EphA3 mutants retained the capacity to associate and co-immunoprecipitate with ADAM10 (Figure 2C, Figure S4B), in agreement with our previous finding that ADAM interacts with EphA3 via specific regions in their extracellular protein domains [9]. However, compared to Wt EphA3 or EphA3-[2YE], binding of the [ΔJXS] and [ΔJXL] mutants to ADAM10 was notably reduced, supporting our hypothesis that approximation of the EphA3 kinase domain to the plasma membrane imparts steric obstruction to ADAM10 binding that is relieved during JM domain unfolding. Also, binding to ADAM10 of “kinase-dead” [2YE-KM] was reduced compared to EphA3-[2YE] (Figure 2C), suggesting either that Eph kinase activity may play a role in facilitating the ADAM10 interaction or that JM domain unfolding of the [2YE-KM] mutant is incomplete, as implied from the crystal structure [27].

**Figure 3:**
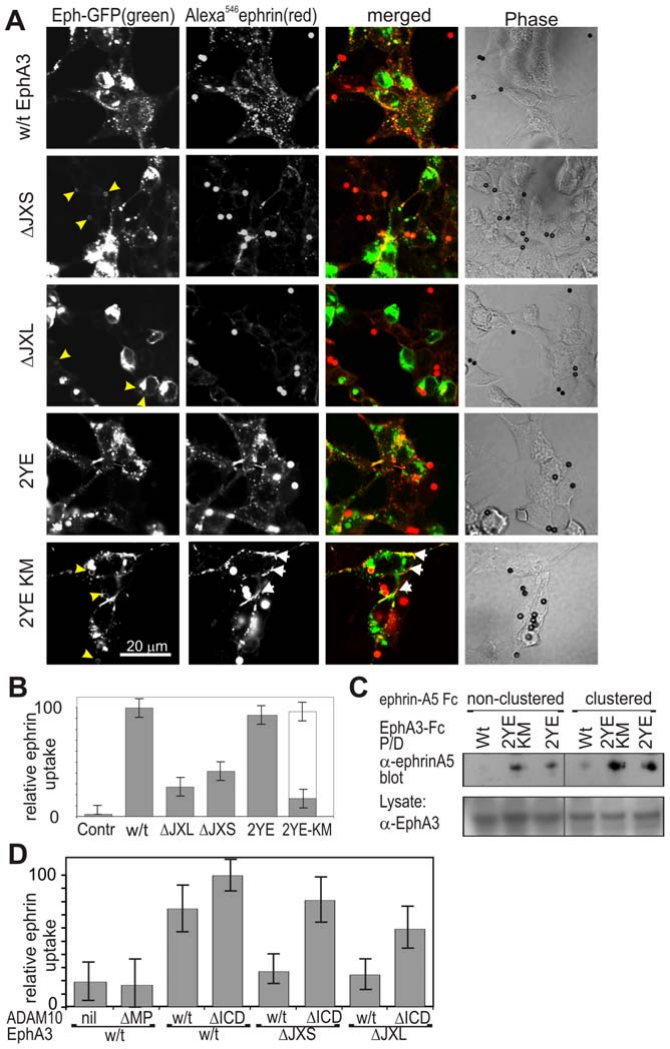
EphA3 JM and kinase domain mutations affect ephrin-A5 shedding and internalisation, respectively.

To test our hypothesis of a conformational switch in the EphA3 JM domain that controls ADAM10 access and ephrin shedding, we compared the ability of Wt and mutant EphA3 receptors to support ADAM-catalysed shedding from ephrin-A5-coated beads and internalisation into EphA3-expressing cells. Confocal microscopy confirmed that indeed both EphA3 JM-truncations significantly affected the capacity to promote ephrin-A5 shedding, whereas the [2YE] mutant behaved comparable to Wt EphA3 (Figure 3A, 3B). Interestingly, kinase-compromised EphA3[2YE-KM] with an unfolded JM domain behaved similar to cytoplasmic-truncated EphA3[ΔICD] (Figure 1C): cell surface ephrin-staining away from the beads revealed ability of this mutant to support ephrin shedding but failure to internalise the shed ligand (Figures 3A, 3B and S5A), also evident when soluble, pre-clustered Alexa^594^ephrin-A5-Fc was used as substrate (Figure S5B). Since EphA3[2YE] bearing an intact kinase but lacking JM tyrosines is internalised normally, this argues for EphA3 endocytosis requiring tyrosine kinase activity and/or phosphorylation, likely of the remaining critical phosphorylation site within the Eph kinase activation-loop [13]. We confirmed the ability to support ephrin-shedding using immunoprecipitation analysis, revealing similar levels of cleaved ephrin-A5 in cultures of EphA3[2YE] and EphA3[2YE-KM] cells (Figure 3C).

These experiments indicate that it is the proximity of the EphA3 kinase domain to the plasma membrane rather than its kinase activity per se that determines if shedding by ADAM10 is inhibited or promoted. For further evidence, and to clarify if there is any contribution from signalling intermediates that may communicate between the Eph and ADAM cytoplasmic domains, as implied from previous studies [4],[5], we compared ephrin-A5 shedding by cells co-expressing EphA3 mutants together with either Wt ADAM10 or with cytoplasmic-truncated ADAM10[ΔICD] (Figures 3D and S6B). ADAM10[ΔICD] would be expected to overcome the steric hindrance exerted by EphA3 JM mutants, compared to full length ADAM10. To avoid the potential ambiguity caused by the presence of endogenous, Wt ADAM10/EphA3 complexes, we performed these experiments in mouse embryonic fibroblasts (MEFs) from ADAM10 KO mice [2] lacking any detectable ADAM10 expression (Figure S6A). Similar to our findings in HEK293T cells, ephrin-A5 shedding in these MEFs was apparent upon co-expression of either Wt or [ΔICD] ADAM10 together with Wt EphA3, and was greatly reduced when either of the EphA3 JM mutants EphA3[ΔJXL] or EphA3[ΔJXS] were expressed with Wt ADAM10. Importantly, co-expression of cytoplasmic-truncated ADAM10[ΔICD] together with EphA3[ΔJXL] or EphA3[ΔJXS] “rescued” the inhibitory effect of the JM positioning of the Eph kinase domain and resulted in shedding comparable to that seen with the Wt ADAM and Eph proteins (Figures 3D and S6B).

Together, these experiments demonstrate that ephrin-A5 shedding by ADAM10 is controlled by steric hindrance exerted by the membrane-proximal EphA3 kinase domain, which prevents the functional interaction with ADAM10 that is needed for efficient substrate (ephrin) cleavage to occur.

### Steric Hindrance as a Conserved Mechanism of ADAM Regulation

In addition to controlling RTK function, ADAMs are key modulators of cell–matrix interactions [29], and ADAM17-catalysed exodomain shedding regulates the function of the leukocyte adhesion protein L-selectin [20]. Of note, L-selectin shedding is blocked by CaM binding to the L-selectin cytoplasmic domain and is promoted by CaM inhibitors [21], indicating a similar regulation by steric hindrance. Intriguingly, these inhibitors also trigger metalloprotease-dependent EGFR signalling [30], further suggesting that steric hindrance, in this case imparted by CaM binding within the EGFR JM region [31], may promote ADAM-catalysed ligand release.

**Figure 4:**
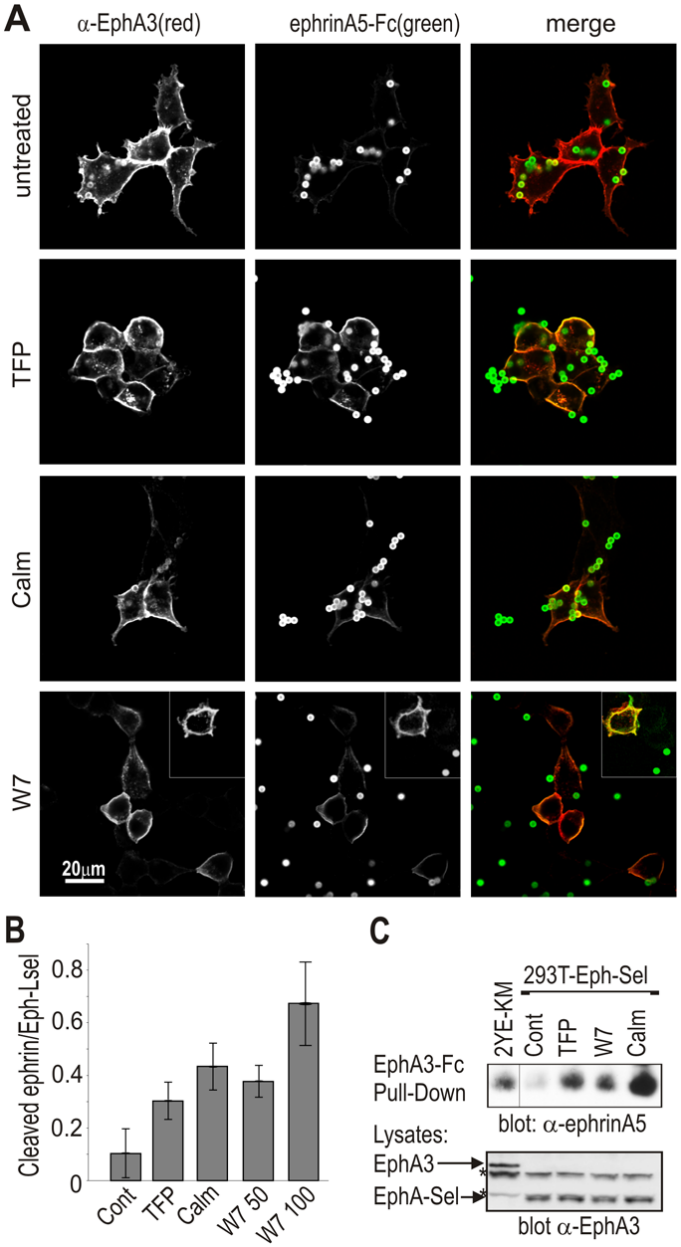
CaM-binding to chimeric EphA3/L-selectin regulates ephrin cleavage. (A) Confocal analysis of ephrin release: EphA3/L-selectin transfected HEK293T cells were pre-treated with CaM inhibitors trifluoperazine dimaleate (TFP, 15 µM), Calm (2 µM), W7 (N-6-Aminohexyl0-5-chloro-1-naphthalenesulfonamide, 50 or 100 µM), or vehicle control before incubation with Alexa^488^ephrin-A5-Fc beads. Cell surface EphA3/L-selectin (Alexa^647^ α-EphA3 antibody, red) and Alexa^488^ephrin-A5 (green) were imaged in fixed cells by confocal microscopy. Insets show cells treated with 100 µM W7. (B) The ratio of ephrin-A5-associated and EphA3/L-selectin associated fluorescence was estimated from images of control or inhibitor-treated cell cultures taken under identical conditions. Mean values are illustrated (*n*>4), with error bars indicating 95% confidence intervals determined by the ANOVA test for multiple comparisons. (C) Biochemical analysis of ephrin release: EphA3/L-selectin transfected HEK293T cells were pre-treated with CaM inhibitors (as in (A), using 50 µM W7) or vehicle control and incubated with ephrin-A5-Fc coated beads. In parallel, EphA3[2YE-KM]-expressing cells were incubated with ephrin-A5-Fc coated beads only. Ephrin-A5 recovered by EphA3-Fc pulldown was immunoblotted with α-ephrin-A5 antibodies. Total lysates were probed for EphA3/L-selectin (Eph-Sel) expression with α-EphA3 antibodies (bottom). * indicates non-relevant proteins recognised by α-EphA3 antibodies in total lysates.

To test the hypothesis that a bulky protein domain at the JM position would impair a productive ADAM/EphA3 alignment, the EphA3 cytoplasmic domain was replaced with that of L-selectin. We surmised that CaM-loaded EphA3/L-selectin could not effectively promote ephrin cleavage, while conversely inhibition of CaM binding to this chimeric receptor using CaM inhibitors should favour ADAM10 association and ephrin-A5 cleavage. Control experiments confirmed inhibition of CaM binding (Figure S7A) and increased ADAM10 association (Figure S7B). Indeed, shedding from ephrin-A5-Fc coated beads was markedly higher in inhibitor-treated than in untreated cells expressing EphA3/L-selectin (Figure 4A, 4B). Furthermore, immunoblotting of cleaved ephrinA5-Fc from cultures of EphA3/L-selectin cells (Figure 4C) confirmed shedding in CaM-inhibitor-treated cells but not in control cells, at levels that are comparable to those observed in EphA3[2YE-KM] cell cultures (Figure 4C). Likewise, engineering of inactivating mutations into the CaM-binding domain [21] (EphLsel EE) notably increased the capacity of these cells to support ephrin shedding as compared to Wt EphA3-L-selectin cells (Figure S7C). Of note, in these experiments ephrin-A5 labelled the cell membrane but was not internalised into cells with EphA3/L-selectin, confirming the need for the intact EphA3 cytoplasmic domain for endocytosis. We confirmed CaM inhibitor-induced shedding also of cell bound ephrin-A5, using co-cultures of green-fluorescent protein (GFP)-ephrin-A5-expressing and EphA3/L-selectin-expressing cells (Figure S8A). In agreement with these imaging experiments, immunoblotting of cleaved GFP-ephrin-A5 recovered from GFP-ephrin-A5-expressing cells that had been co-cultured with EphA3/L-selectin-cells revealed CaM inhibitor-induced shedding, which is absent in inhibitor-treated control cells (Figure S8B). Thus, CaM binding to the EphA3/L-selectin protein effectively regulates shedding of ephrin-A5, further demonstrating that ADAM10 activity is controlled by steric constraints in the JM region of a (chimeric) EphA3 mutant that is devoid of tyrosine kinase– and kinase-dependent signalling activity.

### Activation of EphA3 Is Accompanied by Extension of the ICD

**Figure 5:**
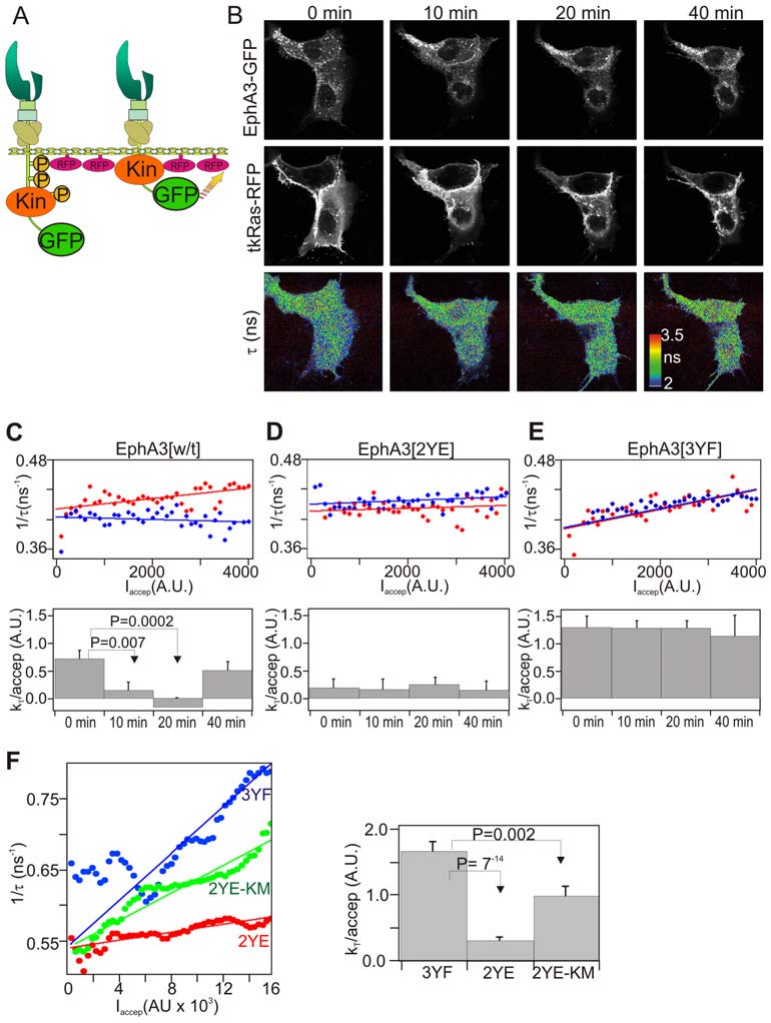
FLIM analysis shows EphA3 activation accompanies extension of the cytoplasmic domain. (A) Schematic of the FRET-assay; FRET (yellow arrow) reflects the proximity between the GFP on the EphA3 C-terminus and ^tkRas^RFP on the inner plasma membrane (Kin, kinase domain). (B) Confocal FLIM time-series of EphA3-GFP in ^tkRas^RFP co-transfected COS7 cells at indicated times (min) after ephrin-A5 stimulation. Upper row: EphA3-GFP fluorescence intensity images. Middle row: ^tkRas^RFP fluorescence intensity images. Lower row: fluorescence lifetime images of EphA3-GFP; the colour bar inset indicates the fluorescence lifetime range in ns. (C) Upper panel: 2D-histograms of fluorescence rate τ^−1^ versus ^tkRas^RFP acceptor intensities for 0 min (red) and 20 min (blue) of the EphA3-GFP confocal FLIM series. Linear fitting of the time series 2D-histograms are indicated by solid lines. Lower panel: acceptor normalized energy transfer rate, *k_T_*/acceptor, for selected time points after EphA3 stimulation as derived from the slope of the fits to the 2D-histograms. (D) Upper panel: 2D-histograms of τ^−1^ ^tkRas^RFP acceptor intensities for 0 min (red) and 20 min (blue) of a 2YE-EphA3-GFP confocal FLIM time series after ephrin-A5 stimulation (see also Figure S6F). Lower panel: acceptor normalized energy transfer rate, *k_T_*/acceptor, of the time series as in (C). (E) Upper panel: 2D-histograms of τ^−1^ ^tkRas^RFP acceptor intensities for 0 min (red) and 20 min (blue) of a 3YF-EphA3-GFP confocal FLIM time series after ephrin-A5 stimulation. Lower panel: acceptor normalized energy transfer rate, *k_T_*/acceptor, of the time series as in (C). (F) Left panel: 2D-histograms of fluorescence rate, τ^−1^, versus acceptor intensities for EphA3 [2YE], [3YF], and [2YE-KM] obtained from at least 16 fluorescence lifetime/acceptor intensity images obtained with wide-field frequency-domain FLIM. Right panel: linear fitting of these data showed a significant difference between the slopes (*k_T_*/acceptor) for EphA3-GFP-[2YE] and EphA3-GFP-[3YF].

To this point our analysis strongly argues for the notion that the “relaxed” and “constrained” conformations of active and inactive EphA3, respectively, would direct functional or dysfunctional alignment of ADAM10 with EphA3. To examine in intact cells, if indeed activation uncoils the EphA3-kinase domain away from the plasma membrane, we developed a Förster resonance energy transfer (FRET) imaging approach [32] that is sensitive to the distance between the EphA3-COOH (C)-terminus and the plasma membrane. Here, we used fluorescence lifetime imaging microscopy (FLIM) to monitor FRET between EphA3-GFP and the inner membrane leaflet of Cos7 cells labelled with membrane-targeted ^tkRas^RFP (red-fluorescent protein) (Figure 5A) [33].

Confocal FLIM analysis of live cells activated with clustered ephrin-A5-Fc revealed that fluorescence lifetimes (τ) of cell-surface EphA3-GFP increased (showing reduced FRET) 10–20 min after stimulation (Figure 5B, 5C and Figure S9A, S9B), indicating a drop of the cytoplasmic domain from a membrane-proximal to a membrane-distal conformation. The receptor population with increased fluorescence lifetimes (activated EphA3) returned to pre-stimulation levels at 40 min, likely reflecting de-phosphorylation of activated EphA3. However, energy transfer from GFP (donor) to ^tkRas^RFP (acceptor) will not only depend on their distance but also on the local acceptor density that may vary as a function of time and space in a FLIM time-lapse series. To account for this, the FRET rate (*k_T_*) per acceptor density (*k_T_*/acceptor) that does not depend on the concentration of acceptors in the plasma membrane [34] needs to be determined. We estimated this parameter from the slopes of a linear fit to the fluorescence rates (τ^−1^)–acceptor intensity (I_accep_) 2D-histograms of the confocal images at selected time points (Figure 5C). Ephrin-A5 stimulation of EphA3 resulted in a significant decrease in *k_T_*/acceptor values (Figure 5C), indicating an increased GFP-RFP distance. The maximal decrease in the *k_T_*/acceptor ratio was observed after 20 min stimulation and was followed by partial recovery of the FRET efficiency to that seen with inactive EphA3. By comparison, a confocal FLIM time series of ephrin-A5 stimulated cells expressing constitutively active EphA3[2YE]-GFP or inactive EphA3[3YF]-GFP, containing Phe-replacements of all critical tyrosine residues (Figure 5D, 5E) [16],[27] yielded no significant change in the *k_T_*/acceptor slopes, indicating that in this case stimulation does not notably change the distance between GFP and RFP (Figure 5D, 5E). The lower *k_T_*/acceptor value for EphA3[2YE]-GFP and the higher value for EphA3[3YF]-GFP as compared to non-stimulated EphA3[wt]-GFP are consistent with constitutively extended and constitutively constrained conformations, respectively, of these receptor mutants.

We note that single cell FLIM analysis of the Wt EphA3 conformation during stimulation exhibits considerable variance, reflecting different mixtures of active and inactive receptor populations at each spatially resolvable volume element in the image. We therefore compared the constitutive conformations of kinase active EphA3[2YE]-GFP with that of inactive EphA3[3YF]-GFP. We computed cumulative 2D-histograms of fluorescence rates versus acceptor intensities for the [2YE]-, [3YF]-, and [2YE-KM]-mutants using fluorescence lifetime/acceptor intensity images of cells for each of the mutant receptors (Figure 5F) obtained with wide-field frequency-domain FLIM (Figure S9C [35]). The energy transfer rate, *k_T_*/acceptor, was calculated from the slope of a linear fit to the fluorescence rate—acceptor intensity 2D-histograms in which the intercept was set to the measured fluorescence rate of the donor (GFP) in the absence of acceptor. The EphA3[2YE]-GFP mutant exhibited significantly (*p* = 7×10^−14^) lower FRET efficiencies (as apparent from *k_T_*/acceptor, right panel, Figure 5F) than the inactive EphA3[3YF]-GFP mutant, indicating its extension from the plasma membrane and confirming the conformational change of the Wt EphA3 ICD observed upon stimulation of live cells. The FRET efficiency of EphA3[2YE-KM]-GFP was in between these extremes, suggesting that kinase-dead Eph with a flexible JM domain adopts an intermediate position between a fully extended and constrained cytoplasmic domain, as previously suggested [27]. The FRET rate (*k_T_*) is proportional to the fourth power of the distance between the donor chromophore and acceptor plane [34]. From the fourth power root of the ratio of *k_T_*/acceptor of EphA3[3YF]-GFP and EphA3[2YE]-GFP (Figure 5F), we can thus estimate that the C-terminus of relaxed ([2YE]) EphA3-GFP is 1.36±0.06 times further away from the plasma membrane than that of constrained ([3YF]) EphA3-GFP.

### Electron Tomography of Quantum Dot (Qdot) Labelled EphA3 Confirms Increased Span of the Active EphA3 ICD

To examine at increased resolution the change in the span between the plasma membrane and the carboxy-terminus of the latent and activated receptor, we used electron microscopy (EM) to image the EphA3 C-terminus that had been labelled with streptavidin-conjugated Qdots (SA-Qdots [36]). We achieved the site-specific targeting with SA-Qdots by engineering onto the EphA3 C-terminus a biotin acceptor peptide (AP), which can be specifically biotinylated using the *E. coli* biotin ligase (BirA) [37].

**Figure 6:**
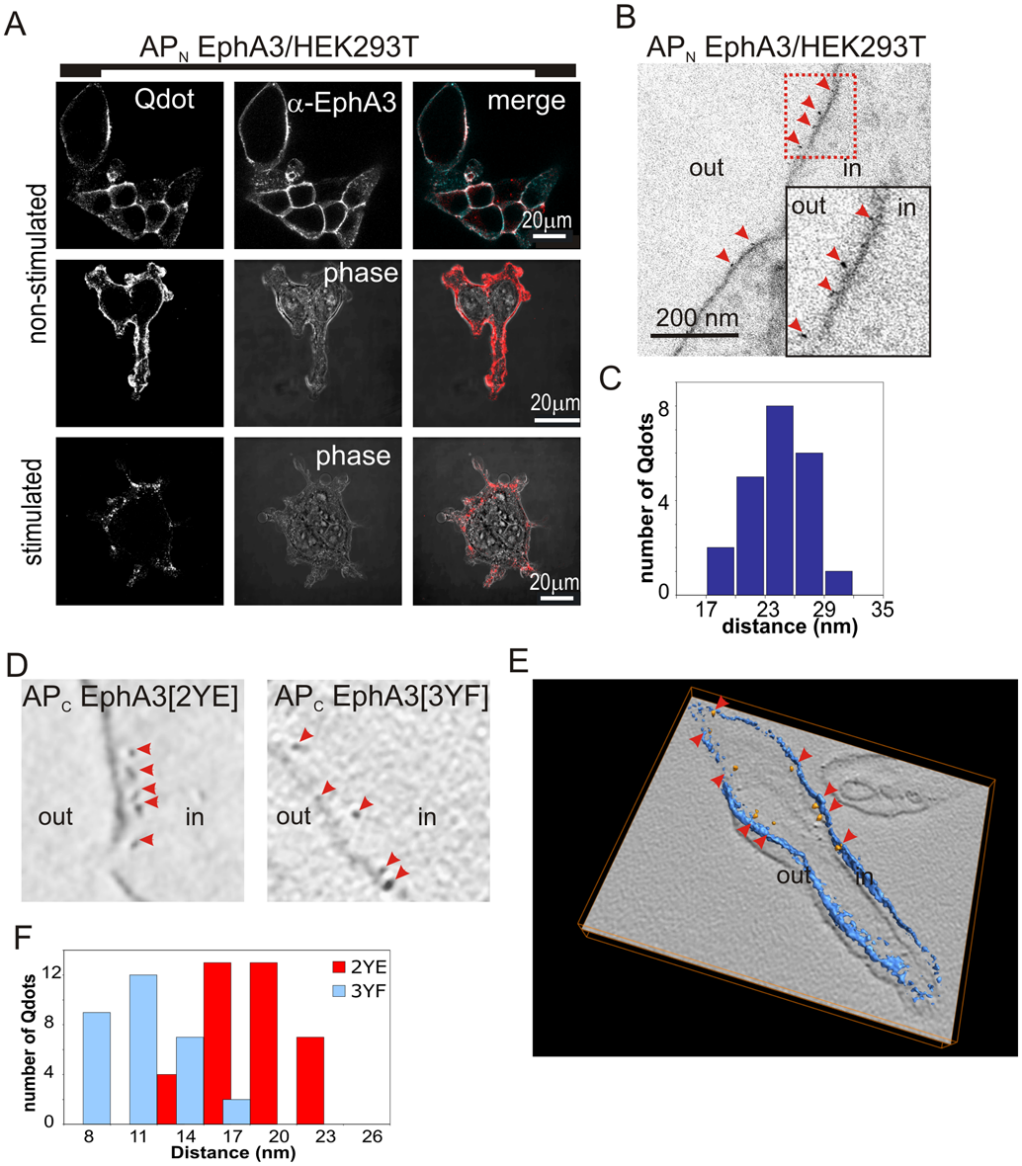
EM of Qdot labelled EphA3 reveals the molecular span to the plasma membrane. (A) Confocal microscopic images of AP_N_ EphA3/HEK293T cells, biotinylated with recombinant biotin ligase (BirA) and labelled with SA-Qdots^605^. Cells were left non-stimulated (top and middle panel) or ephrin stimulated (bottom panel). To show the specificity of binding of the SA-Qdots to EphA3, samples were co-stained with an EphA3-specific monoclonal antibody. Qdot staining is shown in red in the merged images. (B) EM image of biotinylated AP_N_ EphA3/HEK293T cells labelled with SA-Qdots^605^. Arrowheads mark EphA3-tethered Qdots on the outer cell membrane; insert: enlarged section of the (red) boxed area. (C) Histogram depicting distances between Qdots bound to the NH_2_terminus of EphA3 and the plasma membrane. Distances were measured from EM images of biotinylated AP_N_ EphA3/HEK293T cells that were incubated with SA-Qdots^605^ as shown in (B). (D) SA-Qdot^605^ micro-injected cells expressing AP_C_-EphA3[2YE] and [3YF] as indicated. Each image represents a 10 nm thick computational slice after 3D reconstruction from EM tomography. Arrow heads indicate Qdots. (E) 3D reconstruction of an entire tilt series of images of a cell expressing AP_C_-EphA3[3YF] (plasma membrane, blue; Qdots, yellow; and marked by red arrowheads). A corresponding movie is included as supporting information (Video S1). (F) Histogram showing the cytoplasmic span of inactive versus active EphA3. Membrane-Qdot distances were measured in cells expressing biotinylated AP_C_-EphA3[3YF] (blue) or AP_C_-EphA3[2YE] (red) that had been labelled with micro-injected SA-Qdots^605^. Qdot/plasma membrane distances are plotted at 3 nm intervals (3YF, *n* = 30; 2YE, *n* = 37). Kolmogorov-Smirnov (KS) and *t* statistical tests suggested a highly significant (*p*<0.001) difference between the two datasets.

We validated the feasibility of the approach by binding SA-Qdots to an NH_2_-terminal AP-tagged and biotinylated EphA3 (AP_N_-EphA3). Confocal microscopy revealed that SA-Qdot staining of AP_N_-EphA3 cells correlated with anti-EphA3 staining and shifted to a cytoplasmic compartment upon ephrin-stimulation (Figure 6A), suggesting intact endocytosis of Eph-signaling clusters [13]. EM of these cells revealed Qdots at the outer cell surface at discernible distances from the plasma membrane (Figure 6B). Considering that a reasonably broad range of estimates (17–29 nm) likely reflects flexibility of the AP-tag linker region, this provides an apparent distance between Qdots and plasma membrane of ∼24 nm (Figure 6C).

In order to image the span of the EphA3 cytoplasmic domain we analysed cells co-expressing a cytoplasmic form of BirA together with EphA3, AP-tagged at the C-terminus (AP_C_-EphA3). Microinjection of SA-Qdots into AP_C_-EphA3-expressing cells and sectioning of non-permeabilised cells allowed EM of EphA3-bound Qdots at the intact plasma membrane (Figure 6D). We used electron tomography with alignment, reconstruction, and segmentation of 3D images (Figure 6E) to estimate Qdot/membrane distances. For AP_C_-EphA3[3YF] cells (Figure 6D, right panel), these ranged from 8–18 nm, with an average span from the membrane of approximately 12 nm (12.32+/−3.1 nm, Figure 6F). Expression of Wt EphA3 or of EphA3[2YE] rapidly leads to perturbation of the plasma membrane due to EphA3 activation and endocytosis (Figure S10) and prevented assignment of Qdot/membrane distances with and without ephrin stimulation. To allow comparison of data from EM tomography with our FRET analysis, we therefore analysed AP_C_-EphA3[2YE], representing EphA3 with relaxed JM regions, while co-expressing the clathrin-assembly protein AP180 to block endocytosis [38] of activated receptors in these cells (Figure 6D, left panel). The distribution of Qdot positions in these cells was clearly different to AP_C_-EphA3[3YF] cells, suggesting for activated EphA3[2YE] an average span of approximately 19 nm (19.37+/−3.8 nm, Figure 6F), approximately 1.6±0.5 times wider than that of inactive [3YF]EphA3. This relative distance increase is consistent with that determined by FLIM (above) and confirms a notable extension of the activated receptor away from the plasma membrane.

## Discussion

Intracellular regulation of ADAM sheddases is known to control the release of transmembrane growth factor precursors and the activation of corresponding growth factor receptors. It was identified as a cause for EGF receptor transactivation almost a decade ago [17],[19]. However, the mechanism linking kinase and sheddase activities has remained elusive since its inception. We have now elucidated a previously unrecognized conformational switch that is embedded in the cytoplasmic domains of Eph receptors and ADAMs and controls ADAM-function and Eph signalling.

Furthermore, marking the EphA3 C-terminus with fluorescent (GFP) and electron-dense (Qdots) tags allowed for the first time to demonstrate in live cells at molecular resolution that receptor activation and tyrosine phosphorylation triggers a measurable shift of the kinase domain away from the plasma membrane. The increased span of activated EphA3 that we estimated by FLIM and EM tomography is consistent with partial extension of the 64-residue (G_569_ – N_633_) receptor JM domain with a maximal theoretical (β-sheet) span of ∼20 nm. While it would seem formally possible that extension of the activated EphA3 ICD reflects an unfolding of the linker connecting the kinase with the C-terminal SAM domain, recently elucidated crystal structures of the EphA3 ICD argue against this possibility [28]: in the structures of the active and of the inactive form, this C-terminal linker is tethered to the base of the kinase, indicating that the C-terminal part of the EphA3 cytoplasmic domain maintains a rigid, kinase-associated configuration.

**Figure 7:**
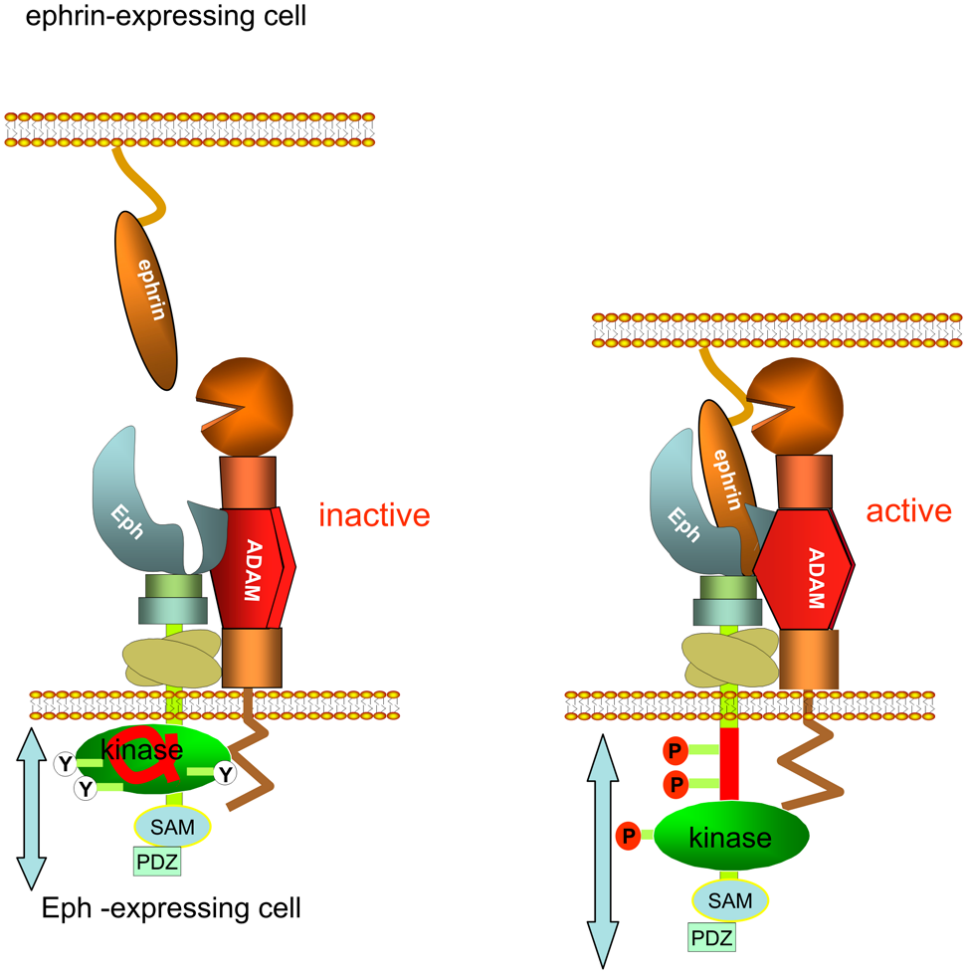
Model for activation-mediated release of the membrane-proximal Eph kinase domain promoting productive ADAM10 alignment and ephrin shedding. The (helical) JM segment (red) of the unligated Eph receptor is tethered to the small (N-terminal) lobe of the kinase [24], keeping the kinase domain (green) in an inactive, membrane-proximal conformation. Ephrin binding, Eph clustering (for simplicity only one Eph/ephrin pair is illustrated instead of a cluster), activation, and auto-phosphorylation result in release of the tyrosine-phosphorylated JM segment into a dynamic disordered protein fold [25] that allows extension of the kinase domain and the Eph C-terminus away from the membrane. ADAM10, constitutively associated with the receptor, can then bind a new site formed by the Eph/ephrin complex via the ADAM10 substrate recognition motif, which in turn mediates the correct orientation of the protease domain for ephrin cleavage [7]. The stoichiometry of the ADAM10/Eph/ephrin complex remains to be elaborated. Importantly, this productive alignment of ADAM10 and Eph RTK relies on a RTK configuration where its kinase domain—exerting steric hindrance for ADAM10 association—is removed from the plasma membrane.

Together, our data suggest a model (Figure 7) where the inactive, membrane-proximal receptor kinase domain obstructs the productive alignment with ADAM10 that is necessary for effective ephrin cleavage. This alignment of the ADAM and Eph extracellular domains relies on a shift of the activated kinase domain away from the membrane and on docking of the ADAM10 substrate-recognition site to the high-affinity Eph/ephrin complex [9]. Such a conformational switch provides rapid and precise control of ADAM10-sheddase activity and challenges the relevance of signalling intermediates that are thought to be involved in the control of ADAM10 sheddase activity [4],[5]:

- Our finding that cytoplasmic truncation of ADAM10 or of EphA3 does not affect ephrin shedding confirms previous studies revealing that phorbol ester-stimulated shedding by ADAM17 is not effected by deletion of its cytoplasmic domain [22].
- We further show that EphA3 JM mutations that permanently raise the kinase domain close to the plasma membrane significantly reduce ADAM10 association and ephrin shedding, while mutations that cause constitutive extension promote ephrin cleavage. This holds true irrespective of kinase activity, as kinase-inactive but conformationally “relaxed” EphA3[2YE-KM] supports shedding, while EphA3[ΔJXS] with an active but membrane-proximal kinase does not.
- Importantly, we demonstrate that inhibition of shedding by the membrane-proximal Eph kinase domain occurs only with intact ADAM10, but is effectively rescued with cytoplasmic-truncated ADAM10 as sheddase: This indicates that it is indeed the relief from a steric clash between the membrane-proximal receptor domain and the ADAM10 cytoplasmic tail that provides the molecular switch that allows productive shedding to occur.

These findings have considerable implications for the understanding of Eph signalling: Currently it is established that contacts between Eph and ephrin-expressing cells that fail to activate robust Eph phosphorylation will lead to cell spreading and cell-cell adhesion while cell-contact induced Eph activation and phosphotyrosine signalling result in cell rounding and cell segregation [11],[12],[39]. Studies demonstrating the critical role of ADAM10-catalysed ephrin shedding for cell repulsion revealed that interaction with a cleavage-resistant ephrin mutant leads to persisting Eph/ephrin contacts but does not prevent cell rounding (axon collapse) [8], suggesting independent—but tightly synchronised—processes during cell repulsion. They further imply that the cell-biological consequence of an Eph/ephrin contact is determined only during assembly of the Eph/ephrin complex: Thus, synchronisation of cell rounding and segregation requires a molecular switch that rapidly relays the signalling competence of the Eph kinase to the protease controlling ephrin shedding.

We now have elaborated the molecular mechanics of this relay in which kinase-active and kinase-inactive Eph receptors adopt distinct protein configurations that allow productive and unproductive association with ADAM10, respectively. Previously, we demonstrated that processed, catalytically active ADAM10 lacking the inhibitory pro-domain [5] is associated with EphA3 also in the absence of ephrin-A5 contact [9]. In the current context this implies that ADAM10 is “on standby” to release Eph-bound ephrin from interacting cells in the moment EphA3 becomes tyrosine-phosphorylated and adopts a conformation that allows ADAM10 alignment for optimal substrate access. Such a mechanism provides for the synchronised Eph-triggered cell rounding and segregation that is observed during cell-cell repulsion.

A host of stimuli that promote ADAM-mediated shedding, including most prominently phorbol esters and calcium ionophores, have been described [5]. However, while interaction with signalling proteins, in particular via SH3-binding motifs, have been postulated, it has remained unclear if and how the different agents could modulate ADAM activity [4]. The concept of regulation via cytosolic intermediates has been challenged in particular by the finding that PMA (phorbol-12-myristate-13-acetate) efficiently activates an ADAM17 mutant lacking the ICD [22]. Furthermore, CaM inhibitor and calcium-ionophore-induced shedding of EGFR-ligands by ADAM10 is retained partially by a mutant lacking the ICD [40]. Our findings now reveal an entirely novel concept of RTK/ADAM regulation whereby the conformation of the cytoplasmic domain directly regulates ADAM activity rather than involving intermediate signalling proteins and kinase activity per se.

It is tempting to speculate that steric hindrance represents a conserved mechanism for receptor-regulated ADAM activity. Regulated L-selectin shedding provides an important example of a very different receptor system that may be controlled by a mechanism, where CaM binding to the L-selectin ICD [21] and its ensuing conformational change seem to hinder the ability of ADAM17 to shed L-selectin. Notably, upon protein binding to its target site, CaM changes from an elongated to a globular structure [41] with very similar dimensions (4–5 nm diameter) to the RTK kinase domain. Our observation that CaM-binding to an EphA3/L-selectin chimeric receptor can also regulate ADAM10 cleavage of ephrins would seem to confirm steric hindrance as a mechanism regulating Eph-associated ADAM activity and to suggest this as a more widely conserved concept of ADAM regulation. For example, steric hindrance could also explain the regulation of ADAM activity by other RTKs such as the EGFR, which binds CaM within the JM region [31] and controls ADAM-facilitated ligand shedding in an activation dependent manner [5]. Indeed, EGFR signalling can be triggered in a metalloprotease-dependent manner by CaM inhibitors [30], preventing CaM access to a binding site within the EGFR JM region [31].

Interestingly, our data suggest that ephrin cleavage and its receptor-mediated internalisation may be controlled separately, since EphA3 with “relaxed” JM but inactive kinase domain effectively supports ephrin cleavage but is not internalised. While details of Eph endocytosis mechanisms remain to be elaborated, this finding is consistent with signalling-dependent RTK endocytosis potentially involving the ubiquitin ligase Cbl [42]. Importantly, effective internalisation of the EphA3 2YE mutant suggests that the JM tyrosines may not be essential for this endocytic signalling mechanism.

Lastly, we have developed a novel imaging strategy, which bridges the gap between structural and cell-biological imaging, to provide functional information for individual proteins in whole cells at molecular resolution, and which could have applications in a wide range of cell-biological systems. Our integrated FLIM- and EM-based analyses reveal that Eph RTK activation triggers, in addition to tyrosine phosphorylation, a measurable extension of the ICD towards the cytosol. The previously unforeseen functional consequences of this conformational change for downstream Eph signalling and the regulation of ADAM10 activity are likely to have important implications for the understanding of ADAM-regulated biological processes in development and disease.

## Material and Methods

### Expression Constructs

Inactive EphA3 was made by substitution of residue K653 to M in EphA3-GFP [13]. Insertion into bovine ADAM10-HA [9] of a KpnI restriction site at C698 and removal of the ICD (retaining the C-terminal HA tag) yielded ADAMΔcyto. For EphA3-L-selectin, a NheI restriction site at EphA3 G565 together with annealed L-selectin ICD oligonucleotides (forward: 5′CTAGGAGATTAAAAAAAGGCAAGAAATCCAAGAGAAGTATGAATGACCC-ATATTAA; reverse: 5′CTAGTTAATATGGGTCATTCATACTTCTCTTGGATTTCTT-GCCTTTTTTTAATCTC) were inserted, with a terminal stop codon. For EphA3-AP_N_ the AP-tag [37] was inserted after the EphA3 signal sequence (after Gly_20_), and for EphA3-AP_C_ the AP-tag was inserted into a XmaI site engineered into the EphA3 C-terminus (Val_983_).

### Biochemical Analyses

Cleaved ephrin-A5 was extracted from pooled Protein-A Sepharose-pre-cleared lysates and culture supernatants of cells that had been treated with pre-clustered or non-clustered ephrin-A5-Fc by using EphA3-Fc coupled to Protein-A-Sepharose. Pull-downs were analysed by anti-ephrin-A5 immunoblot. Cleavage of cell-surface ephrin-A5 was assayed in 1-h co-cultures of ephrin-A5-expressing HEK293T cells and EphA3/L-selectin transfected cells by extracting ephrin-A5 from cell lysates with EphA3-Fc coated Protein-A Sepharose. Where indicated, cells were treated prior to ephrin-A5-stimulation with CaM inhibitors trifluoperazine, calmidazolium (Calm), or W7, or metalloprotase inhibitors TAPI1 or GM6001 (Calbiochem). For CaM-co-precipitation EphA3/L-selectin and Wt EphA3 tagged with a biotin AP were biotinylated with BirA [37] and recovered on SA dynabeads. Other co-immunoprecipitation experiments were performed as indicated with anti-ADAM10 mAb (R&D Systems), anti-ADAM10 polyclonal Ab39177 (Abcam), with anti-EphA3 mAb IIIA4 [9] pre-coupled to mini-leak™ agarose (Kem-En-Tec, Copenhagen), and with anti-phosphotyrosine Sepharose (4G10, Upstate Biotechnology). Transient expression of all EphA3 constructs was optimised by transfecting each at four cDNA concentrations and selecting samples with similar expression levels by Western blotting total lysates. Western blotting was performed with antibodies against ephrin-A5 (R&D systems), EphA3 [43], HA (3F10, Roche), ADAM10 (Biogenesis and Abcam Ab39177), phosphotyrosine (4G10, Upstate Biotechnology), and CaM (Upstate Biotechnology).

### Confocal Microscopy

3-channel confocal microscopy was performed by sequential scanning on Olympus FV1000 or Leica SP5 confocal microscopes. Quantitation of internalised ephrin-A5-associated fluorescence was achieved using ImageJ or Metamorph image analysis software by selecting regions of cells to exclude bead-associated fluorescence. Microscopic evaluation of ephrin cleavage by EphA3/L-selectin expressing cells, where ephrin-A5 was not internalised but remained complexed at the plasma membrane, was done by estimating the level of Alexa^488^-ephrin labelling relative to the expression level of the Alexa^647^IIIA4 anti-EphA3 antibody-stained receptor [15] on cell membranes (Figure 4B). Interactions between cell-surface ephrin-A5 and EphA3/L-selectin were analysed using ephrin-A5-GFP transfected cells [9].

### FLIM

Time-domain confocal FLIM was performed in transiently transfected Cos7 cells grown on coverslips or glass bottom dishes (MatTek Corp.). FLIM images were obtained using an Olympus Fluoview 1000 microscope, equipped with a Picoharp 300 photon counting setup (Picoquant, Germany). GFP was excited with a 470 nm diode (Sepia II, Picoquant, Germany). Images of 512×512 pixels were acquired detecting approximately 10^8^ photons. Images of the donor fluorescence decays were processed using the SymPhoTime software package (v4.2, Picoquant) and the calculated average fluorescence lifetime (τ) images are presented in pseudo-colour. The average fluorescence lifetime τ(xy) images were calculated from the parameters (a_1_,a_2_,τ_1_,τ_2_) of a double exponential fit of the fluorescence decay curves [F(x,y,t)] in each pixel:
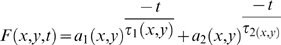
(1.1)At pixel x, y the average fluorescence lifetime is:
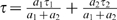
(1.2)τ^−1^-acceptor intensity (I_a_) 2D-histograms were computed from the confocal FLIM images as described below for wide-field frequency-domain FLIM except that a bin size of 100 counts was used for the acceptor intensity.

For wide-field frequency-domain FLIM (Figure S9) we used an IX70 inverted microscope (Olympus, Japan) equipped with a 100/1.4 NA oil immersion lens, a 476 nm argon laser and narrow-band emission filter (HQ510/20; Chroma) for GFP, a 100-W mercury arc lamp with high Q Cy3 filter set (excitation filter, HQ545/30; dichroic, Q580LP; emission filter, HQ610/75) for RFP, and a dichroic beamsplitter (Q495 LP; Chroma Technology, Brattleboro, VT) and narrow-band emission filter.

Raw FLIM data were processed in IPLab (Scanalytics, Fairfax, VA, USA) to generate a binary mask for the intensity threshold operation data and a mask for the ROI used in background correction of the raw data. Using the raw FLIM data and mask, phase- and modulation lifetime images were generated using scripts written in Python programming language (http://www.python.org) with the Numarray extension for numerical computing (http://www.stsci.edu/resources/software_hardware/numarray) further augmented with low-level routines written in C [44],[45]. A cumulative 2D-histogram of fluorescence lifetime (τ) versus acceptor intensity (I_a_) was generated from the multiple fluorescence phase-lifetime images (≥16 images) and corresponding acceptor intensity images using a bin size of 320 intensity units (arbitrary units). The standard error in the fluorescence lifetimes for each bin was calculated from the averages of all the images. The donor to acceptor energy transfer rate k_T_ normalized to acceptor density (k_T_/acceptor) was obtained from the slope of a linear fit to the τ^−1^−I_a_ (acceptor intensity) 2D-histograms. The τ^−1^−I_a_ 2D-histograms were fitted to a linear equation:
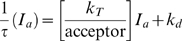
(1.3)in which prior knowledge of the fluorescence lifetime in the absence of acceptor (measured, τ_d_ = 1.96 ns) was used to constrain the intercept, k_d_, to 0.51. The slope k_T_/acceptor is proportional to the energy transfer rate per acceptor yielding 1.9+/−0.16 for EphA3[3YF]-GFP, 0.56+/−0.08 for EphA3[2YE]-GFP and 1.27+/−0.16 for EphA3[2YE-KM]-GFP. The relative distance increase from GFP to the plasma membrane, comparing both conformations, (R_2YE_/R_3YF_) can be calculated from:
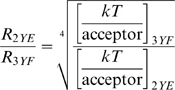
(1.4)yielding a distance increase of GFP to the plasma membrane of 1.36+/−0.06 for EphA3[2YE]-GFP relative to EphA3[3YF]-GFP.

### EM

For EM we biotinylated AP-tagged EphA3 receptors in intact cells using either exogenous or co-transfected biotin ligase (BirA) [37], as indicated, before labelling with SA-Qdots^605^ (Invitrogen). Labelled cells were washed in PBS, fixed in 2.5% Glutaraldehyde, 2% sucrose for 40 min, and prepared on ice for EM by “epon” embedding: cells were rinsed in CaCo buffer (30 min), post-fixed in 2% osmium tetroxide (40 min), washed (water), and stained with 0.5% uranyl acetate (30 min). Fixed, washed cells were dehydrated in graded ethanol solutions, embedded in epon 812 (Serva), and hardened (48 h) at 60°C. Epoxy-embedded blocks were cut into 50 or 250 nm sections (Leica Ultracut S microtome) and mounted on Formvar coated grids. Grids were post-stained with led-citrate (1 min) at room temperature, rinsed with water, and air dried. AP_C_-EphA3 expressing cells, stably co-expressing APc-EphA3 together with a cytoplasmic form of BirA for efficient biotinylation of the AP-tagged EphA3 C-terminus, were either microinjected with Qdots prior to fixation (where indicated) or were fixed in 4% PFA, 0.5% Glutaraldehyde, 2% sucrose (30 min), permeabilised with 0.1% Triton ×100, and incubated with SA-Qdots^605^ for 1 h. Washed samples were then fixed in 2.5% Glutaraldehyde, 2% sucrose, and prepared for EM as described above. This approach partially solubilises the plasma membrane and required computer-assisted assignment of the exact plasma membrane/cytoplasm boundaries.

### Electron Tomography

We collected at room temperature single-axis tilt series of chemically fixed cells at 1–2° angular increment between −67° to +67° using CM200 and Tecnai 30 microscopes (FEI, Eindhoven, The Netherlands) and the Tietz tomography interface (Tietz, Gauting, Germany) for data acquisition. Serial EM images were recorded on 2 k×2 k and 4 k×4 k pixel CCD cameras at a defocus level of −2 µm, with a pixel size at the specimen level of 0.7 nm. We aligned the projection images of the samples using cross-correlation techniques. The merit figure of the aligned tilt-series had a value of approximately 1 nm, indicating no significant shrinkage of the sample. Reconstructions were performed [46] using weighted back-projection algorithms and visualized with isosurface and volume-rendering techniques in the Amira software package (Mercury Computer Systems, San Diego, CA, USA, www.amiravis.com). We de-noised three-dimensional images with nonlinear anisotropic diffusion and semi-automatically segmented those using erosion and dilation operations after roughly segmenting regions of the reconstructions manually. Plasma membranes localisation in the electronic images was semi-automated, with their boundaries determined using dilation and erosion operations. Qdot detection was fully automated according to their size and contrast using thresholding techniques [46].

## Supporting Information

Figure S1 **Tyrosine phosphorylation and ADAM10 association of EphA3 mutants.** (A) Phosphorylation of Wt and mutant EphA3 after incubation with clustered ephrinA5 Fc. HEK293T cell clones stably expressing either Wt or kinase inactive EphA3[K653M], or parental HEK293T cells, were incubated with vehicle control (−), with non-clustered (NC) or clustered (C) ephrinA5-Fc for 15 min prior to lysis. EphA3 immuno-precipitates were analysed by Western blot with anti-phosphotyrosine (α-PY) and lysates with anti-EphA3 antibodies as indicated. (B) The EphA3/ADAM10 association does not require their ICDs. α-HA immunoprecipitates from cells expressing HA-ADAM10, and/or Wt EphA3 or EphA3[ΔICD], were immunoblotted for EphA3 (top) or ADAM10 (bottom); total lysates were probed for EphA3 (right). (U), unprocessed; (P), processed ADAM10. Single exposures of blots are shown with non-relevant lanes removed.
(0.64 MB TIF)

Figure S2 **Inhibition of EphA3ΔICD-dependent ephrin cleavage by metalloprotease inhibitors.** Cells transiently expressing EphA3[ΔICD] were incubated 1 h with the metalloprotease inhibitor GM6001 (10 and 20 µM) or the ADAM-specific inhibitor TAPI1 (50 µM) prior to incubation with Alexa^594^-ephrin-A5-coated beads. After 40 min the cells were placed on ice, stained with anti-EphA3 (IIIA4)-Alexa^647^, fixed and imaged by confocal microscopy.
(4.45 MB TIF)

Figure S3 **Cell surface expression and ephrin binding capacity of EphA3 mutants.** HEK293T cells were transfected with Wt or mutant EphA3-GFP constructs as indicated and analysed for cell surface EphA3 expression by labelling with Alexa^647^-conjugated IIIA4 anti-EphA3 antibody specific for the native EphA3 conformation [13] and with Alexa^594^-conjugated ephrinA5-Fc (ephrinA5-Alexa^594^). Flow cytometric analysis shows cell surface receptor expression (α-EphA3-Alexa^647^) relative to overall expression level (GFP) and to the ability of cells to bind ephrin-A5-Alexa^594^. The fraction of GFP-tagged EphA3 protein on the cell surface was estimated as fraction of GFP-tagged receptor recognised by the anti-EphA3 antibody: all EphA3 ICD mutants are expressed at the plasma membrane and bind the IIIA4 antibody and ephrin-A5 at levels similar to the Wt receptor.
(0.96 MB TIF)

Figure S4 **Phosphotyrosine profile and ADAM10 binding capacity of EphA3 JM mutants.** (A) Phosphotyrosine profile in cells transfected transiently to express Wt EphA3 and JM mutants. HEK293T cells were transfected with expression constructs for Wt EphA3-GFP or derived mutants, as indicated, and cells treated with non-clustered or pre-clustered ephrin-A5 Fc for 10 min. Anti-phosphotyrosine (PY) antibody (4G10) immuno-precipitates from whole cell lysates were probed with anti-PY, and total lysates with anti-EphA3 antibodies, as indicated. Positions on the Western blot corresponding to molecular weights of GFP-EphA3 and IgG (heavy and light chains) are indicated on the left. Phosphorylated protein bands at the GFP-EphA3 position in the left panel are likely due to auto-phosphorylation due to high transient over-expression of the EphA3 constructs in these samples. (B) ADAM10 association with Wt and mutant EphA3. ADAM10 immunoprecipitates and total cell lysates from Wt or mutant (as indicated) EphA3-transfected cells (ephrin-A5-treated) were analysed for EphA3 and ADAM10 by immunoblot. Single exposures of blots are shown with non-relevant lanes removed.
(1.02 MB TIF)

Figure S5 **The extended (active) EphA3 ICD conformation is sufficient for ephrin cleavage, while internalisation requires an intact kinase.** (A) Cells expressing Wt EphA3-GFP, EphA3[2YE]-GFP, or kinase-inactive EphA3[2YE KM]-GFP were incubated with Alexa^594^ephrinA5-coated beads or (B) pre-clustered, soluble Alexa^594^ephrinA5. EphA3-GFP (green) and Alexa^594^ephrin (red) fluorescence in fixed cells was imaged by confocal microscopy. Individual micrographs from fluorescent channels, the merged images, and phase-contrast images are shown. Yellow arrow heads denote areas of sustained interactions between cell surface EphA3 and ephrin-A5 beads. White arrows mark cell-membrane areas with bound- but not internalised Alexa^594^ephrin.
(2.71 MB TIF)

Figure S6 **Removal of the ADAM10 ICD reconstitutes ephrin shedding in cells expressing EphA3 JX mutants.** (A) Confirmation that ADAM10−/− MEFs do not contain detectable ADAM10. Lysates of HEK293Ts, ADAM10−/−, or Wt MEFs were immunoprecipitated with 1, protein A beads alone; or with 2, anti-human specific ADAM10 monoclonal antibodies (RND); or 3, with anti-ADAM10 polyclonal antibodies (Abcam). Immunoprecipitates were immuno-blotted with polyclonal anti-ADAM10 antibodies. U, unprocessed; P, processed ADAM10. (B) ADAM10−/− MEFs transfected with combinations of GFP-tagged EphA3 (Wt, ΔJXS, or ΔJXL) and HA-tagged ADAM10 (Wt, ΔMP, or ΔICD) were incubated with Alexa^594^ephrinA5-coated beads. After 40 min the cells were fixed, permeabilised, and stained with anti-HA and Alexa^647^-labelled secondary antibodies. Images show single-section confocal micrographs, together with the merged images (EphA3-GFP, green; Alexa^594^- ephrin-A5, red; Alexa^647^-anti-HA, blue).
(4.57 MB TIF)

Figure S7 **CaM inhibitors regulate association of EphA3-L-selectin with CaM and with ADAM10 and trigger ephrinA5 shedding by EphA3-L-selectin expressing cells.** (A) CaM inhibitors block association of CaM with EphA3-L-selectin. Cells expressing AP-tagged EphA3/L-selectin or Wt AP-EphA3 were treated with CaM inhibitors trifluoperazine (TFP) or Calm or vehicle control, as indicated. Following biotinylation of AP-tagged receptors, EphA3 complexes were recovered by SA pulldown and analysed by Western blot with anti-CaM and anti-EphA3 antibodies. The positions of Wt EphA3 and of the EphA3/L-selectin fusion protein are indicated. (B) CaM inhibitors modulate the association of EphA3/L-selectin with ADAM10. HEK293T cells expressing EphA3/L-selectin (left panels) or Wt EphA3 (right panels) were pre-treated (30 min) with CaM inhibitors TFP (20 µM), Calm (2 µM), N-6-Aminohexyl0-5-chloro-1-naphthalenesulfonamide (W7, 100 µM), or vehicle control before lysis. ADAM10 immunoprecipitates were analysed by Western blot with anti-EphA3 or anti-ADAM10 antibodies, and total lysates with anti-EphA3 antibodies, as indicated. The graph shows amounts of EphA3/L-selectin (left panels) or EphA3 (right panels) in ADAM10 immunoprecipitates relative to control lanes as determined by densitometry. (C) Mutation of the CaM-binding site in EphA3-L-selectin reduces its ability to support ephrinA5 cleavage. L358E and K359E substitutions, reported to affect CaM binding to the L selectin cytoplasmic domain, were introduced into the EphA3/L selectin chimera to produce EphLsel EE. HEK293T cells, transfected with Wt EphA3-L-selectin or with EphLsel EE were incubated with Alexa^594^-labelled ephrinA5 beads; the capacity to promote ephrin cleavage was monitored by measuring ephrin labelling of the cell membrane. Ephrin labelling relative to receptor expression was determined in 50 regions from five individual micrographs for each sample. The mean+/−SEM are shown in the graph.
(1.48 MB TIF)

Figure S8 **CaM-binding to chimeric EphA3/L-selectin regulates ephrin cleavage from cells.** (A) Microscopic analysis of cleavage of GFP-ephrinA5 from cells. EphA3/L-selectin transfected HEK293T cells were pre-treated (15 min) with CaM inhibitors trifluoperazine (TFP, 15 µM), Calm (2 µM), W7 (50 µM), or vehicle control before incubation (1 h) with cells expressing GFP-ephrinA5. Cell surface EphA3/L-selectin (Alexa^647^ α-EphA3 antibody, red) and GFP-ephrinA5 (green) were imaged in fixed cells by confocal microscopy, micrographs from individual green and red fluorescence channels, and merged images are shown. The outline of GFP-ephrin-A5 expressing cells is indicated (….) for clarity. The open arrow head points at the interface between untreated, EphA3/L-selectin, and GFP-ephrinA5 cells. Yellow arrowheads indicate areas on CaM-inhibitor-treated EphA3/L-selectin cells that are not in direct contact with GFP-ephrin-A5 expressing cells but reveal obvious ephrin staining. (B) Biochemical analysis of GFP-ephrinA5 cleavage from cells. EphA3/L-selectin transfected HEK293T cells were pre-treated as in (A) with TFP, Calm, or vehicle control, then incubated for 1 h with stably transfected ephrinA5/HEK293T cells. Ephrin-A5 was recovered from cell lysates by pulldown with EphA3-Fc beads and detected on Western blot with α-ephrinA5 antibodies. Full-length and cleaved ephrin are indicated. Total lysates were also probed for EphA3/L-selectin expression with α-EphA3 antibodies (bottom). Cleaved ephrin-A5 was quantitated by densitometry.
(2.57 MB TIF)

Figure S9 **FLIM analysis of the EphA3-ICD reveals extension of the activated EphA3 transmembrane domain.** (A) Wide field frequency domain FLIM time-series of EphA3-GFP (green) and ^tkRas^RFP (red) co-transfected COS7 cells at indicated times (min) after ephrin-A5 stimulation. Upper row: EphA3-GFP fluorescence intensity images. Lower row: fluorescence phase lifetime (τ_ϕ_) images of EphA3-GFP colour bar inset indicates the fluorescence lifetime range in ns. Lower image: ^tkRas^RFP fluorescence intensity image. (B) Histograms of GFP phase lifetimes τ_Φ_ calculated on a pixel-by-pixel basis for the cells displayed in (A). (C) Example of GFP phase (τ_Φ_) and modulation (τ_M_) fluorescence lifetime images obtained by FLIM to generate *τ^1^*-acceptor 2D-histograms (Figure 5F) of ^tkRas^RFP-COS7 cells co-expressing Wt, [2YE], [2YE KM], or [3YF] EphA3-GFP. Strong (cytosolic) EphA3 GFP fluorescence was blacked out to exclude areas where the detector was saturated. Cumulative (2D) phase (τ_Φ_) and modulation (τ_M_) fluorescence lifetime histograms of cell populations (right panels) indicate significant fluorescence lifetime differences between EphA3-GFP-[2YE] and EphA3-GFP-[3YF]. (D) The Kolmogorov-Smirnov (KS) test was performed to assess if fluorescence lifetimes of either EphA3-GFP [3YF] or EphA3-GFP [2YE] measured in a large population of tkRasRFP-Cos7 cells are distinct. A highly significant (*p*<0.001) difference between the two datasets suggests a measurable change in the donor (EphA3-GFP)/acceptor (RFP-labelled membrane) distance. The two datasets were entered for the KS test at http://www.physics.csbsju.edu/stats/KS-test.html. (E) Acceptor photobleaching confirms the reliability of fluorescence lifetime imaging to detect FRET. ^tkRas^RFP-transfected (red) Cos7 cells co-expressing EphA3-GFP [3YF] (green) were imaged by FLIM. The images were taken from the same cell before (bottom row) and following (top row) RFP (acceptor) photobleaching. Areas of the cell where the fluorescent acceptor was photobleached show an increase in the GFP lifetime of the donor compared to the nonbleached sample, confirming the fidelity of lifetime imaging to detect FRET.
(2.47 MB TIF)

Figure S10 **EM of Qdot-labelled EphA3.** EM images of fixed AP_C_-EphA3-expressing HEK293T cells without pretreatment (A) or following ephrin stimulation (B), showing plasma membrane disruption and endocytosis, respectively. Cells were fixed, permeabilized, and stained with SA-Qdots 605 prior to EM plastic embedding. Inserts show an enlarged section of the boxed (red) areas, using computer-assisted assignment to delineate the exact plasma membrane/cytoplasm boundary. Arrowheads mark EphA3-tethered Qdots on the inner cell membrane.
(1.01 MB TIF)

Video S1 **EM tomographic reconstruction of Qdot-labelled EphA3-bearing cell membrane.** EM tomography of a protrusion of APC-EphA3 [3YF]-transfected cells, where Qdots label the EphA3 COOH-terminus. The movie shows an iso-surface visualization of the 3D tomographic reconstruction displayed in Figure 6E from any aspect (360°). The plasma membrane is visualized in blue and Qdots are shown in dark yellow.
(0.47 MB AVI)

## Abbreviations

ADAM: A-Disintegrin-And-Metalloprotease
AP_C_: C-terminal Biotin acceptor peptide
BirA: *E. coli* biotin ligase
CaM: calmodulin
Calm: calmidazolium
EGFR: epidermal growth factor receptor
EM: electron microscopy
Ephrin: eph receptor ligand
ephrin-A5-Fc: fusion protein between ephrin-A5 and the human IgG1Fc domain
FLIM: fluorescence lifetime imaging microscopy
FRET: Förster resonance energy transfer
GFP: green-fluorescent protein
HEK293T: transformed human embryonic kidney cells
ICD: intracellular domain
JM: juxtamembrane
MEFs: mouse embryonic fibroblasts
Qdots: quantum dots
RFP: red-fluorescent protein
RTK: receptor tyrosine kinase
SA: streptavidin
SEM: standard error of mean
TFP: trifluoperazine
Wt: wild type
