# Supplementary figures and images for: Cytoplasmic Relaxation of Active Eph Controls Ephrin Shedding by ADAM10
Source: PLoS Biol. 2009 Oct 13;7(10):e1000215. doi: 10.1371/journal.pbio.1000215 (PMC2753297; doi:10.1371/journal.pbio.1000215)

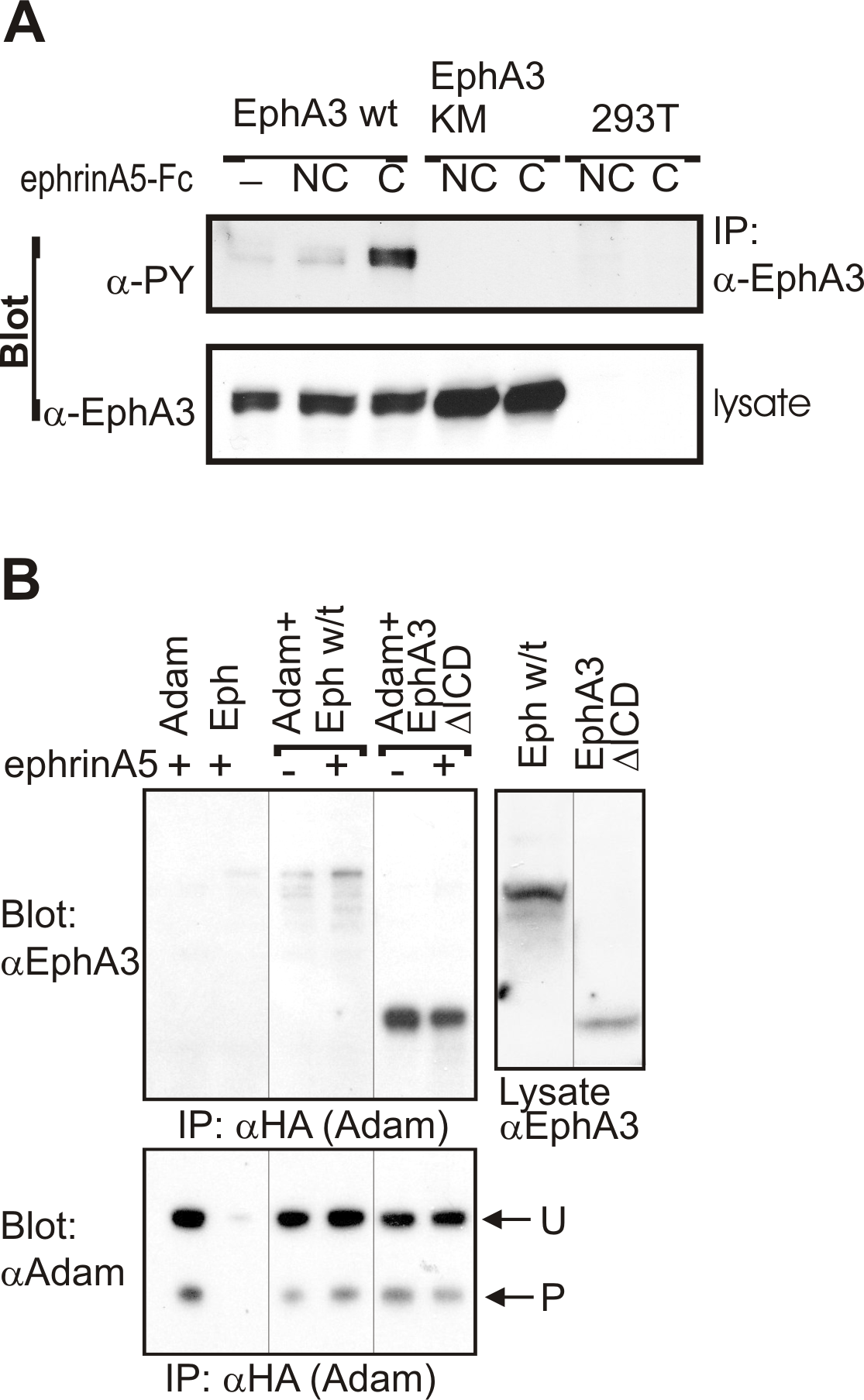

Supplement: Figure S1 — Tyrosine phosphorylation and ADAM10 association of EphA3 mutants. (A) Phosphorylation of Wt and mutant EphA3 after incubation with clustered ephrinA5 Fc. HEK293T cell clones stably expressing either Wt or kinase inactive EphA3[K653M], or parental HEK293T cells, were incubated with vehicle control (−), with non-clustered (NC) or clustered (C) ephrinA5-Fc for 15 min prior to lysis. EphA3 immuno-precipitates were analysed by Western blot with anti-phosphotyrosine (α-PY) and lysates with anti-EphA3 antibodies as indicated. (B) The EphA3/ADAM10 association does not require their ICDs. α-HA immunoprecipitates from cells expressing HA-ADAM10, and/or Wt EphA3 or EphA3[ΔICD], were immunoblotted for EphA3 (top) or ADAM10 (bottom); total lysates were probed for EphA3 (right). (U), unprocessed; (P), processed ADAM10. Single exposures of blots are shown with non-relevant lanes removed. (0.64 MB TIF) [file pbio.1000215.s001.tif]

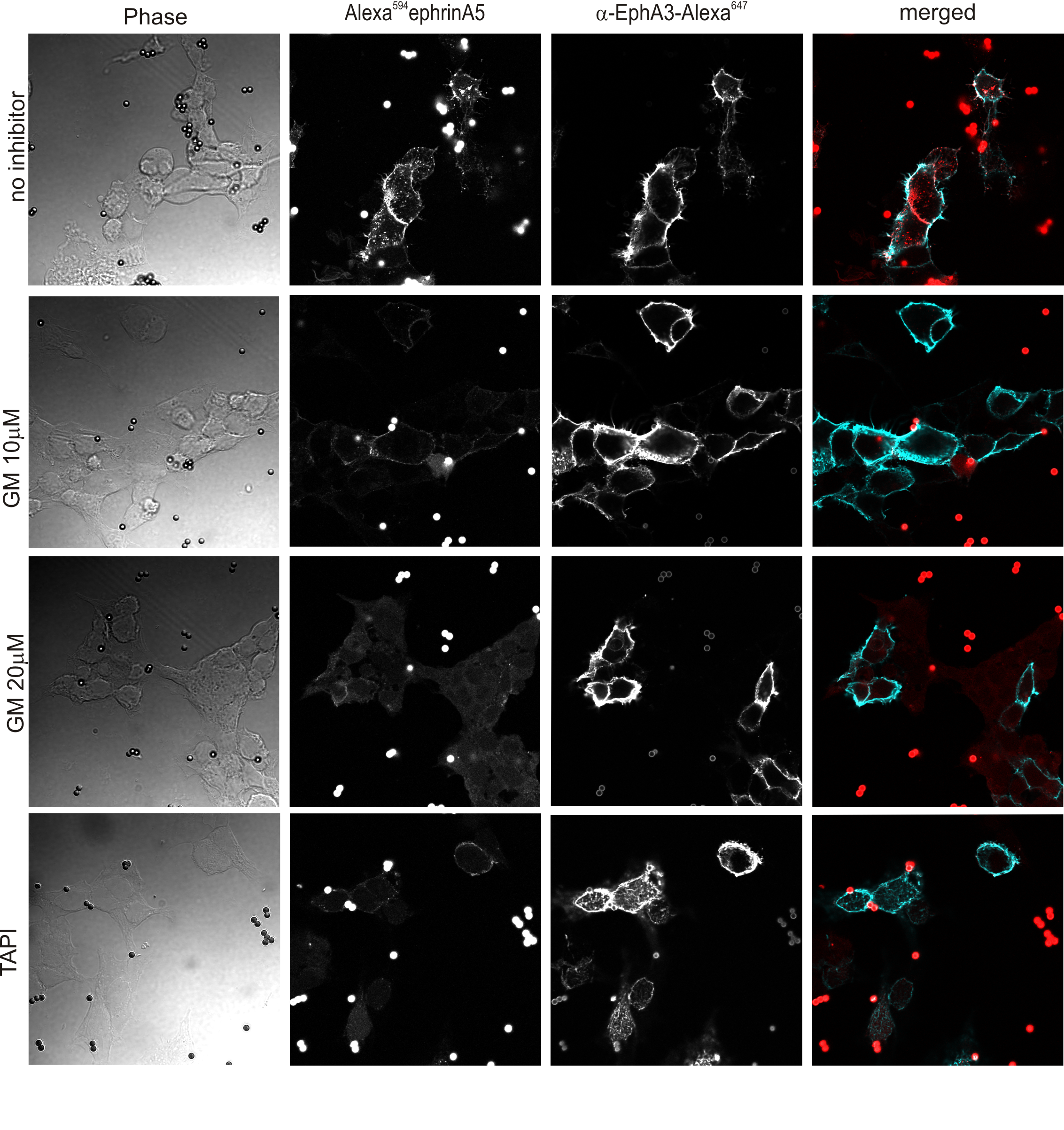

Supplement: Figure S2 — Inhibition of EphA3ΔICD-dependent ephrin cleavage by metalloprotease inhibitors. Cells transiently expressing EphA3[ΔICD] were incubated 1 h with the metalloprotease inhibitor GM6001 (10 and 20 µM) or the ADAM-specific inhibitor TAPI1 (50 µM) prior to incubation with Alexa594-ephrin-A5-coated beads. After 40 min the cells were placed on ice, stained with anti-EphA3 (IIIA4)-Alexa647, fixed and imaged by confocal microscopy. (4.45 MB TIF) [file pbio.1000215.s002.tif]

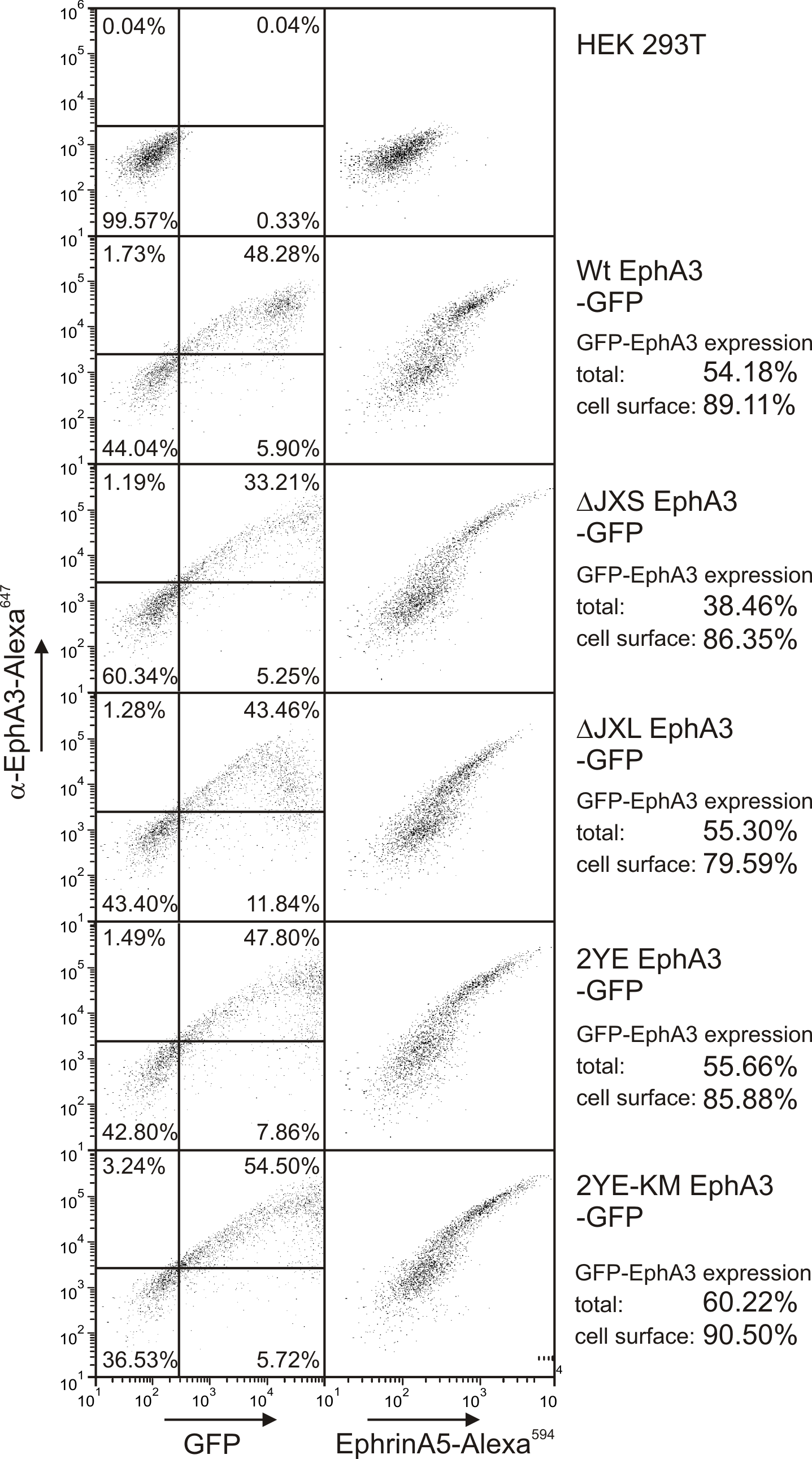

Supplement: Figure S3 — Cell surface expression and ephrin binding capacity of EphA3 mutants. HEK293T cells were transfected with Wt or mutant EphA3-GFP constructs as indicated and analysed for cell surface EphA3 expression by labelling with Alexa647-conjugated IIIA4 anti-EphA3 antibody specific for the native EphA3 conformation [13] and with Alexa594-conjugated ephrinA5-Fc (ephrinA5-Alexa594). Flow cytometric analysis shows cell surface receptor expression (α-EphA3-Alexa647) relative to overall expression level (GFP) and to the ability of cells to bind ephrin-A5-Alexa594. The fraction of GFP-tagged EphA3 protein on the cell surface was estimated as fraction of GFP-tagged receptor recognised by the anti-EphA3 antibody: all EphA3 ICD mutants are expressed at the plasma membrane and bind the IIIA4 antibody and ephrin-A5 at levels similar to the Wt receptor. (0.96 MB TIF) [file pbio.1000215.s003.tif]

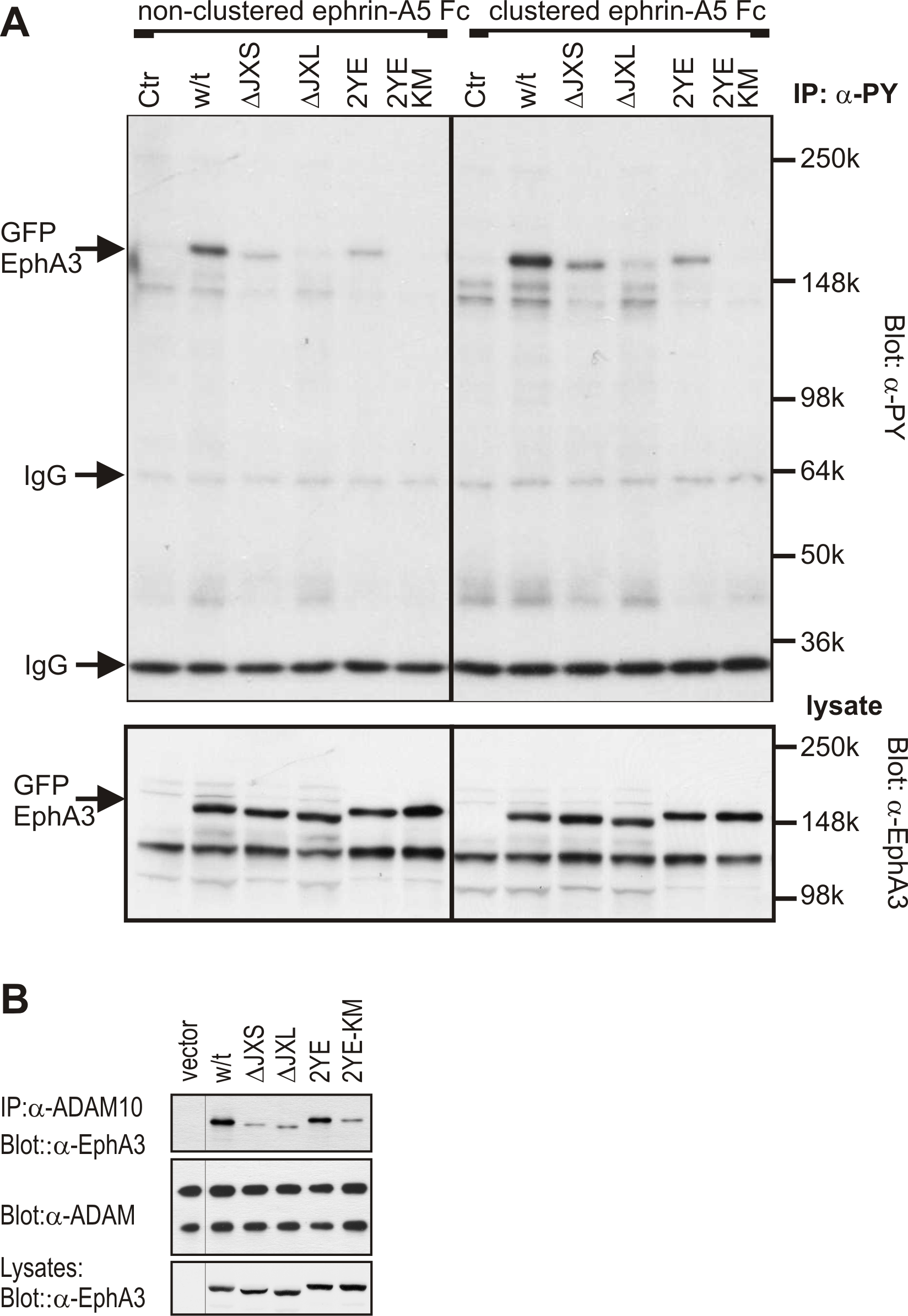

Supplement: Figure S4 — Phosphotyrosine profile and ADAM10 binding capacity of EphA3 JM mutants. (A) Phosphotyrosine profile in cells transfected transiently to express Wt EphA3 and JM mutants. HEK293T cells were transfected with expression constructs for Wt EphA3-GFP or derived mutants, as indicated, and cells treated with non-clustered or pre-clustered ephrin-A5 Fc for 10 min. Anti-phosphotyrosine (PY) antibody (4G10) immuno-precipitates from whole cell lysates were probed with anti-PY, and total lysates with anti-EphA3 antibodies, as indicated. Positions on the Western blot corresponding to molecular weights of GFP-EphA3 and IgG (heavy and light chains) are indicated on the left. Phosphorylated protein bands at the GFP-EphA3 position in the left panel are likely due to auto-phosphorylation due to high transient over-expression of the EphA3 constructs in these samples. (B) ADAM10 association with Wt and mutant EphA3. ADAM10 immunoprecipitates and total cell lysates from Wt or mutant (as indicated) EphA3-transfected cells (ephrin-A5-treated) were analysed for EphA3 and ADAM10 by immunoblot. Single exposures of blots are shown with non-relevant lanes removed. (1.02 MB TIF) [file pbio.1000215.s004.tif]

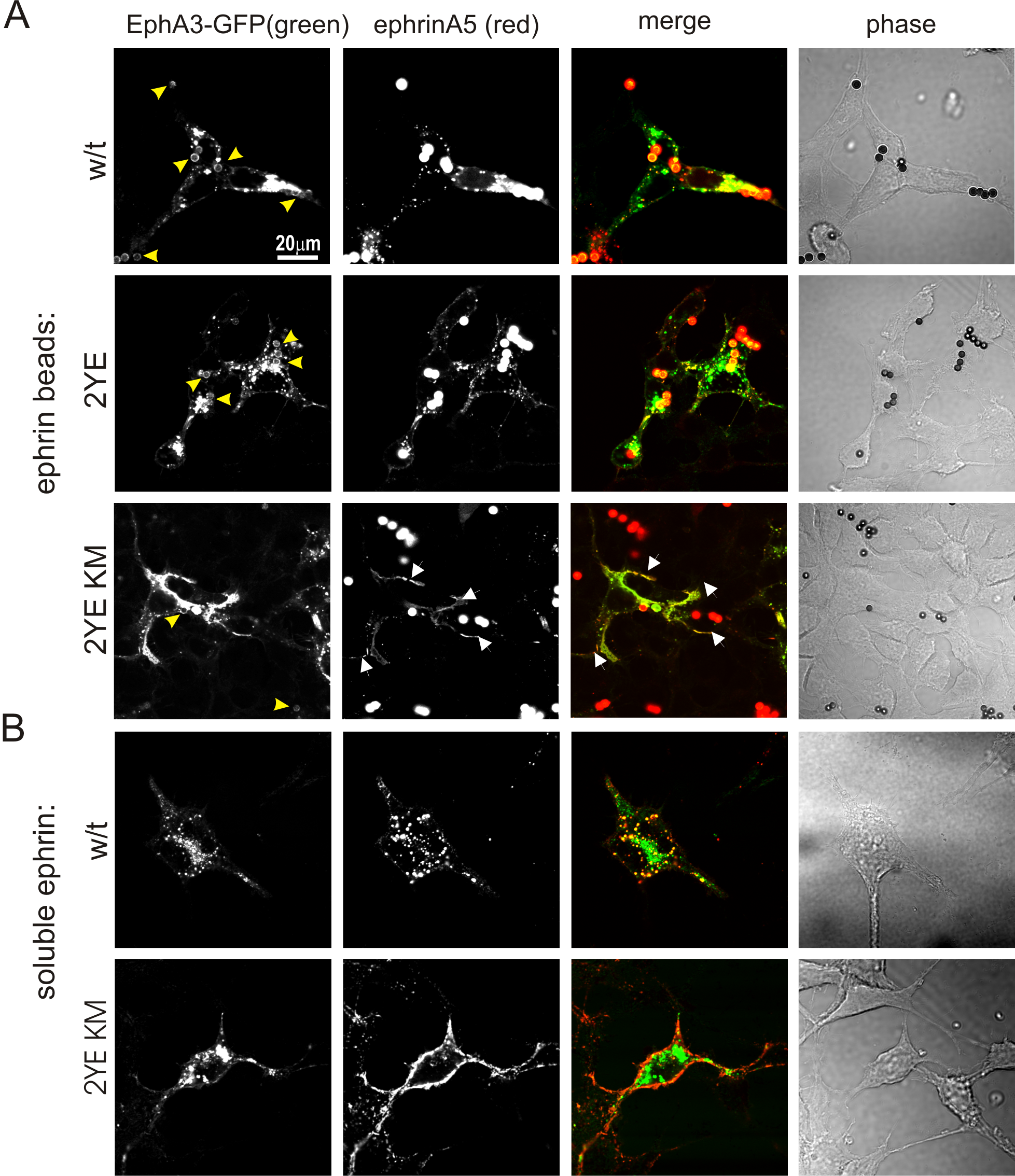

Supplement: Figure S5 — The extended (active) EphA3 ICD conformation is sufficient for ephrin cleavage, while internalisation requires an intact kinase. (A) Cells expressing Wt EphA3-GFP, EphA3[2YE]-GFP, or kinase-inactive EphA3[2YE KM]-GFP were incubated with Alexa594ephrinA5-coated beads or (B) pre-clustered, soluble Alexa594ephrinA5. EphA3-GFP (green) and Alexa594ephrin (red) fluorescence in fixed cells was imaged by confocal microscopy. Individual micrographs from fluorescent channels, the merged images, and phase-contrast images are shown. Yellow arrow heads denote areas of sustained interactions between cell surface EphA3 and ephrin-A5 beads. White arrows mark cell-membrane areas with bound- but not internalised Alexa594ephrin. (2.71 MB TIF) [file pbio.1000215.s005.tif]

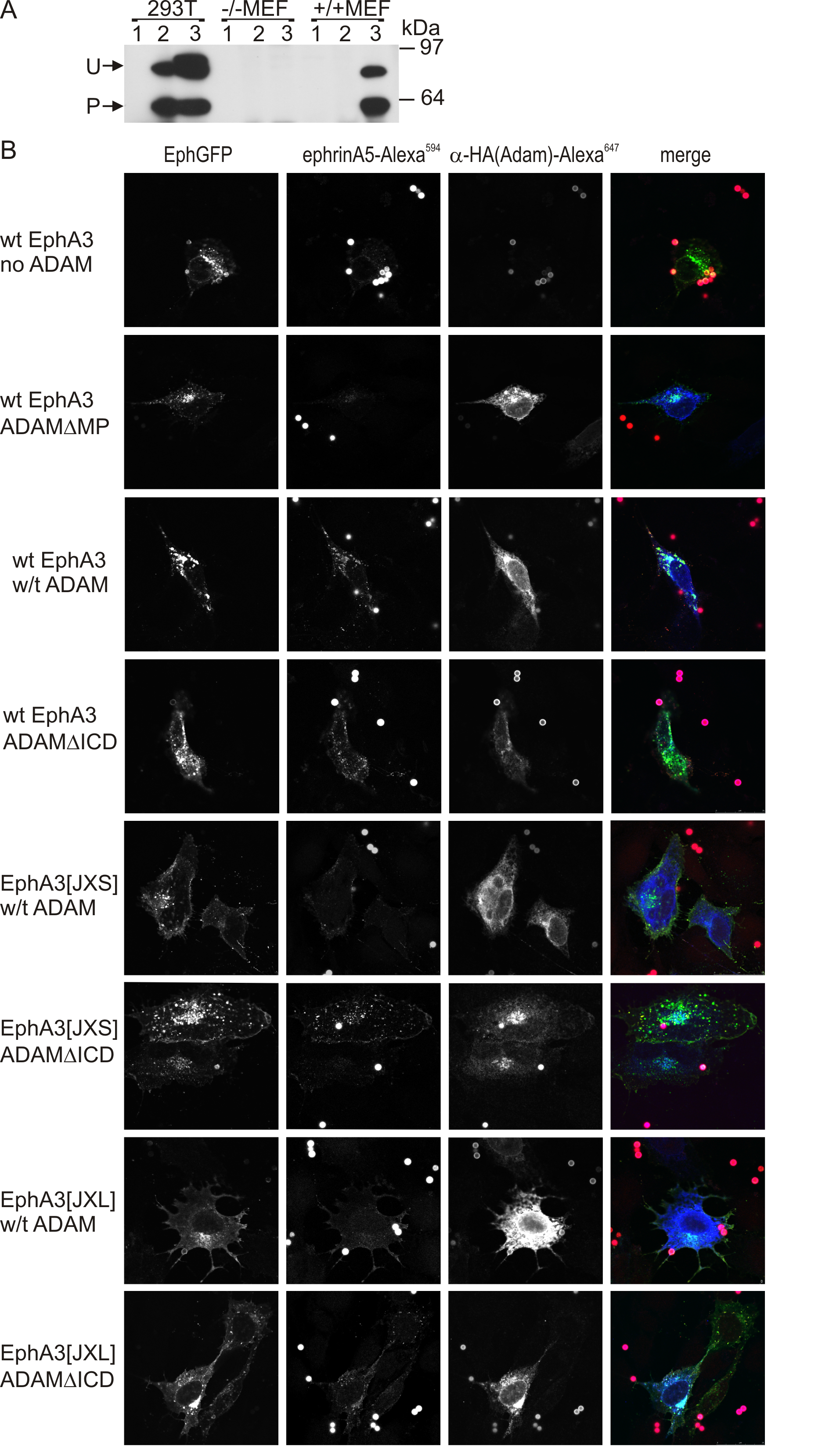

Supplement: Figure S6 — Removal of the ADAM10 ICD reconstitutes ephrin shedding in cells expressing EphA3 JX mutants. (A) Confirmation that ADAM10−/− MEFs do not contain detectable ADAM10. Lysates of HEK293Ts, ADAM10−/−, or Wt MEFs were immunoprecipitated with 1, protein A beads alone; or with 2, anti-human specific ADAM10 monoclonal antibodies (RND); or 3, with anti-ADAM10 polyclonal antibodies (Abcam). Immunoprecipitates were immuno-blotted with polyclonal anti-ADAM10 antibodies. U, unprocessed; P, processed ADAM10. (B) ADAM10−/− MEFs transfected with combinations of GFP-tagged EphA3 (Wt, ΔJXS, or ΔJXL) and HA-tagged ADAM10 (Wt, ΔMP, or ΔICD) were incubated with Alexa594ephrinA5-coated beads. After 40 min the cells were fixed, permeabilised, and stained with anti-HA and Alexa647-labelled secondary antibodies. Images show single-section confocal micrographs, together with the merged images (EphA3-GFP, green; Alexa594- ephrin-A5, red; Alexa647-anti-HA, blue). (4.57 MB TIF) [file pbio.1000215.s006.tif]

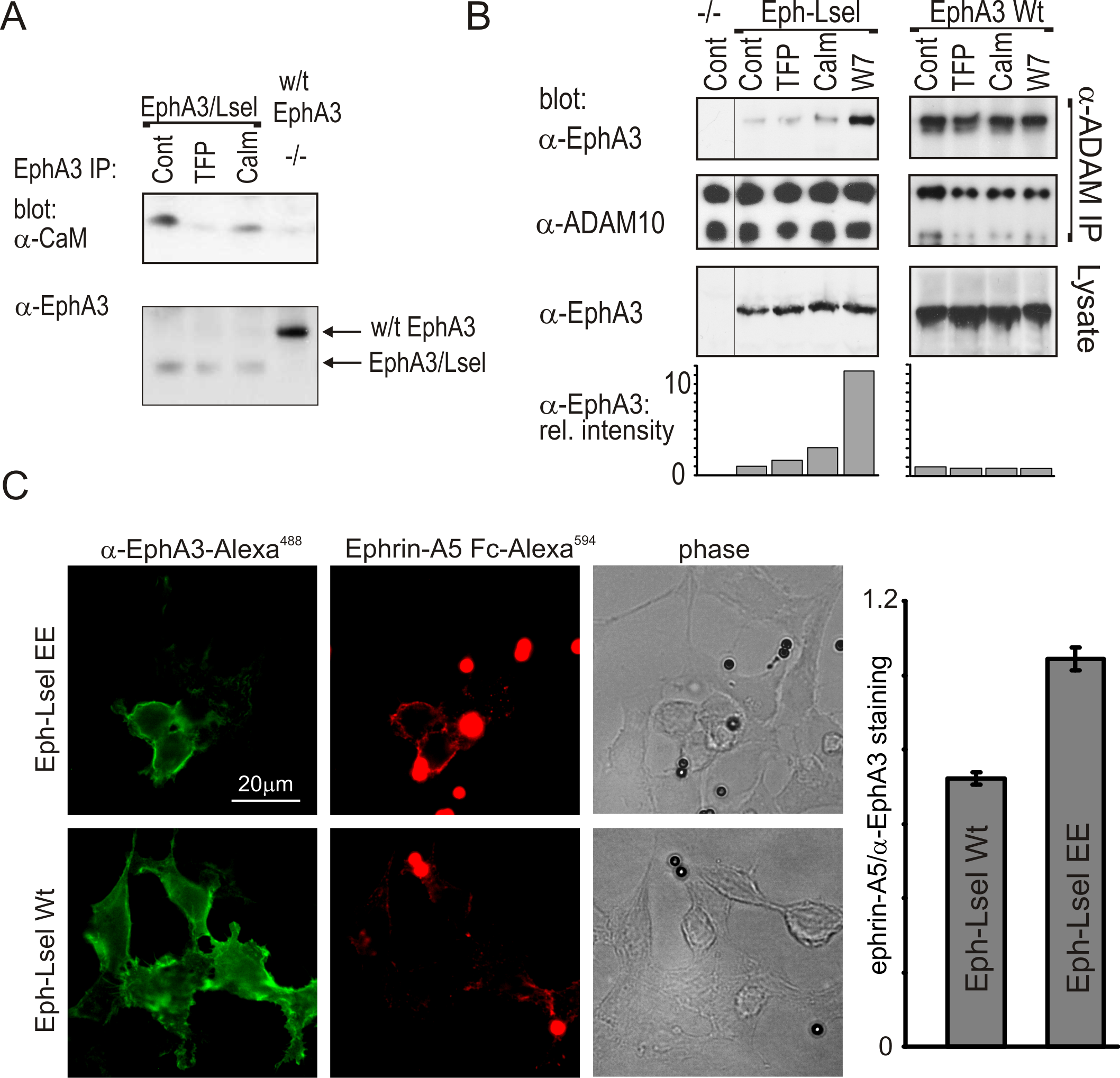

Supplement: Figure S7 — CaM inhibitors regulate association of EphA3-L-selectin with CaM and with ADAM10 and trigger ephrinA5 shedding by EphA3-L-selectin expressing cells. (A) CaM inhibitors block association of CaM with EphA3-L-selectin. Cells expressing AP-tagged EphA3/L-selectin or Wt AP-EphA3 were treated with CaM inhibitors trifluoperazine (TFP) or Calm or vehicle control, as indicated. Following biotinylation of AP-tagged receptors, EphA3 complexes were recovered by SA pulldown and analysed by Western blot with anti-CaM and anti-EphA3 antibodies. The positions of Wt EphA3 and of the EphA3/L-selectin fusion protein are indicated. (B) CaM inhibitors modulate the association of EphA3/L-selectin with ADAM10. HEK293T cells expressing EphA3/L-selectin (left panels) or Wt EphA3 (right panels) were pre-treated (30 min) with CaM inhibitors TFP (20 µM), Calm (2 µM), N-6-Aminohexyl0-5-chloro-1-naphthalenesulfonamide (W7, 100 µM), or vehicle control before lysis. ADAM10 immunoprecipitates were analysed by Western blot with anti-EphA3 or anti-ADAM10 antibodies, and total lysates with anti-EphA3 antibodies, as indicated. The graph shows amounts of EphA3/L-selectin (left panels) or EphA3 (right panels) in ADAM10 immunoprecipitates relative to control lanes as determined by densitometry. (C) Mutation of the CaM-binding site in EphA3-L-selectin reduces its ability to support ephrinA5 cleavage. L358E and K359E substitutions, reported to affect CaM binding to the L selectin cytoplasmic domain, were introduced into the EphA3/L selectin chimera to produce EphLsel EE. HEK293T cells, transfected with Wt EphA3-L-selectin or with EphLsel EE were incubated with Alexa594-labelled ephrinA5 beads; the capacity to promote ephrin cleavage was monitored by measuring ephrin labelling of the cell membrane. Ephrin labelling relative to receptor expression was determined in 50 regions from five individual micrographs for each sample. The mean+/−SEM are shown in the graph. (1.48 MB TIF) [file pbio.1000215.s007.tif]

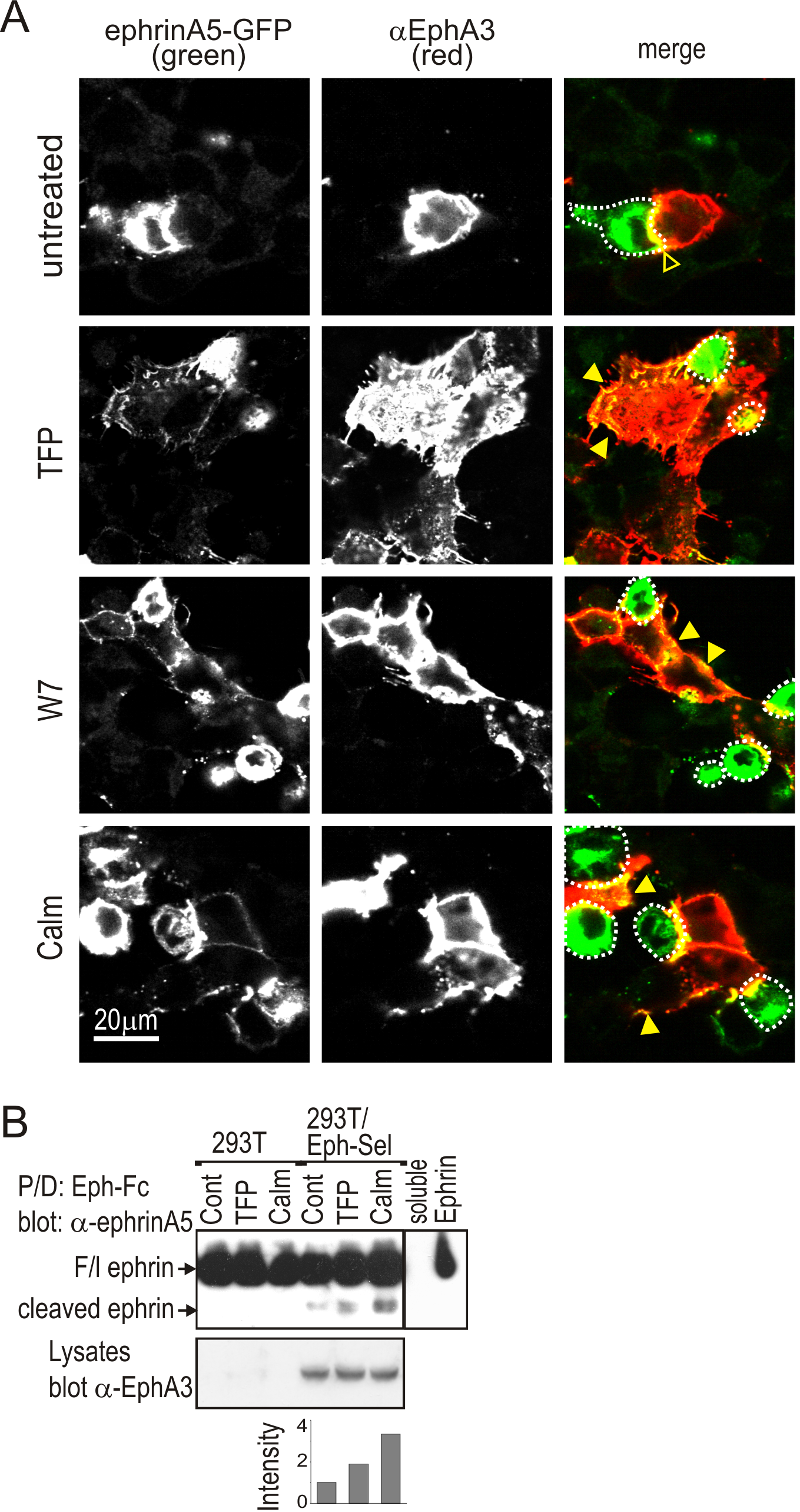

Supplement: Figure S8 — CaM-binding to chimeric EphA3/L-selectin regulates ephrin cleavage from cells. (A) Microscopic analysis of cleavage of GFP-ephrinA5 from cells. EphA3/L-selectin transfected HEK293T cells were pre-treated (15 min) with CaM inhibitors trifluoperazine (TFP, 15 µM), Calm (2 µM), W7 (50 µM), or vehicle control before incubation (1 h) with cells expressing GFP-ephrinA5. Cell surface EphA3/L-selectin (Alexa647 α-EphA3 antibody, red) and GFP-ephrinA5 (green) were imaged in fixed cells by confocal microscopy, micrographs from individual green and red fluorescence channels, and merged images are shown. The outline of GFP-ephrin-A5 expressing cells is indicated (….) for clarity. The open arrow head points at the interface between untreated, EphA3/L-selectin, and GFP-ephrinA5 cells. Yellow arrowheads indicate areas on CaM-inhibitor-treated EphA3/L-selectin cells that are not in direct contact with GFP-ephrin-A5 expressing cells but reveal obvious ephrin staining. (B) Biochemical analysis of GFP-ephrinA5 cleavage from cells. EphA3/L-selectin transfected HEK293T cells were pre-treated as in (A) with TFP, Calm, or vehicle control, then incubated for 1 h with stably transfected ephrinA5/HEK293T cells. Ephrin-A5 was recovered from cell lysates by pulldown with EphA3-Fc beads and detected on Western blot with α-ephrinA5 antibodies. Full-length and cleaved ephrin are indicated. Total lysates were also probed for EphA3/L-selectin expression with α-EphA3 antibodies (bottom). Cleaved ephrin-A5 was quantitated by densitometry. (2.57 MB TIF) [file pbio.1000215.s008.tif]

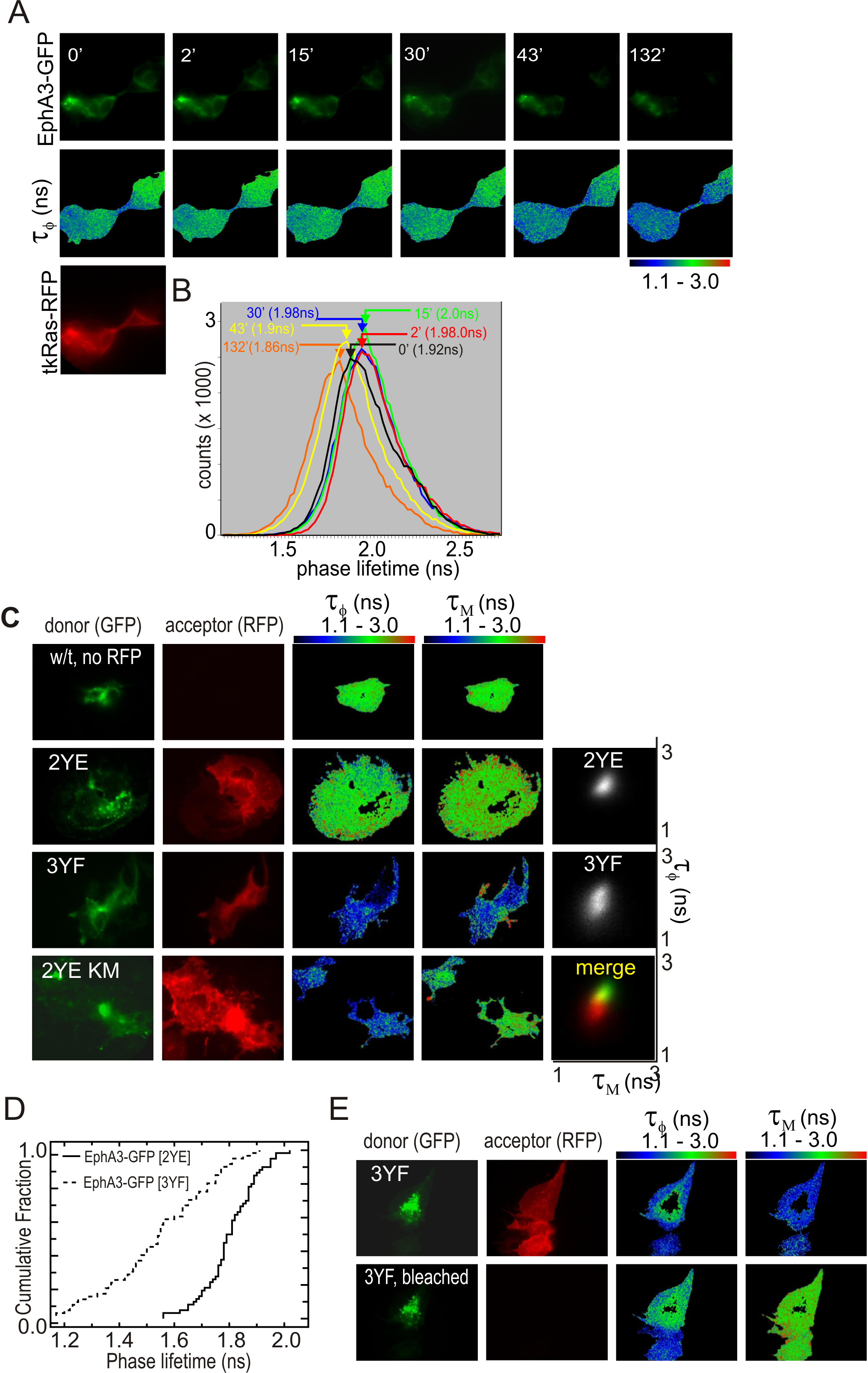

Supplement: Figure S9 — FLIM analysis of the EphA3-ICD reveals extension of the activated EphA3 transmembrane domain. (A) Wide field frequency domain FLIM time-series of EphA3-GFP (green) and tkRasRFP (red) co-transfected COS7 cells at indicated times (min) after ephrin-A5 stimulation. Upper row: EphA3-GFP fluorescence intensity images. Lower row: fluorescence phase lifetime (τϕ) images of EphA3-GFP colour bar inset indicates the fluorescence lifetime range in ns. Lower image: tkRasRFP fluorescence intensity image. (B) Histograms of GFP phase lifetimes τΦ calculated on a pixel-by-pixel basis for the cells displayed in (A). (C) Example of GFP phase (τΦ) and modulation (τM) fluorescence lifetime images obtained by FLIM to generate τ1-acceptor 2D-histograms (Figure 5F) of tkRasRFP-COS7 cells co-expressing Wt, [2YE], [2YE KM], or [3YF] EphA3-GFP. Strong (cytosolic) EphA3 GFP fluorescence was blacked out to exclude areas where the detector was saturated. Cumulative (2D) phase (τΦ) and modulation (τM) fluorescence lifetime histograms of cell populations (right panels) indicate significant fluorescence lifetime differences between EphA3-GFP-[2YE] and EphA3-GFP-[3YF]. (D) The Kolmogorov-Smirnov (KS) test was performed to assess if fluorescence lifetimes of either EphA3-GFP [3YF] or EphA3-GFP [2YE] measured in a large population of tkRasRFP-Cos7 cells are distinct. A highly significant (p<0.001) difference between the two datasets suggests a measurable change in the donor (EphA3-GFP)/acceptor (RFP-labelled membrane) distance. The two datasets were entered for the KS test at http://www.physics.csbsju.edu/stats/KS-test.html. (E) Acceptor photobleaching confirms the reliability of fluorescence lifetime imaging to detect FRET. tkRasRFP-transfected (red) Cos7 cells co-expressing EphA3-GFP [3YF] (green) were imaged by FLIM. The images were taken from the same cell before (bottom row) and following (top row) RFP (acceptor) photobleaching. Areas of the cell where the fluorescent acceptor was photobleached [file pbio.1000215.s009.tif]

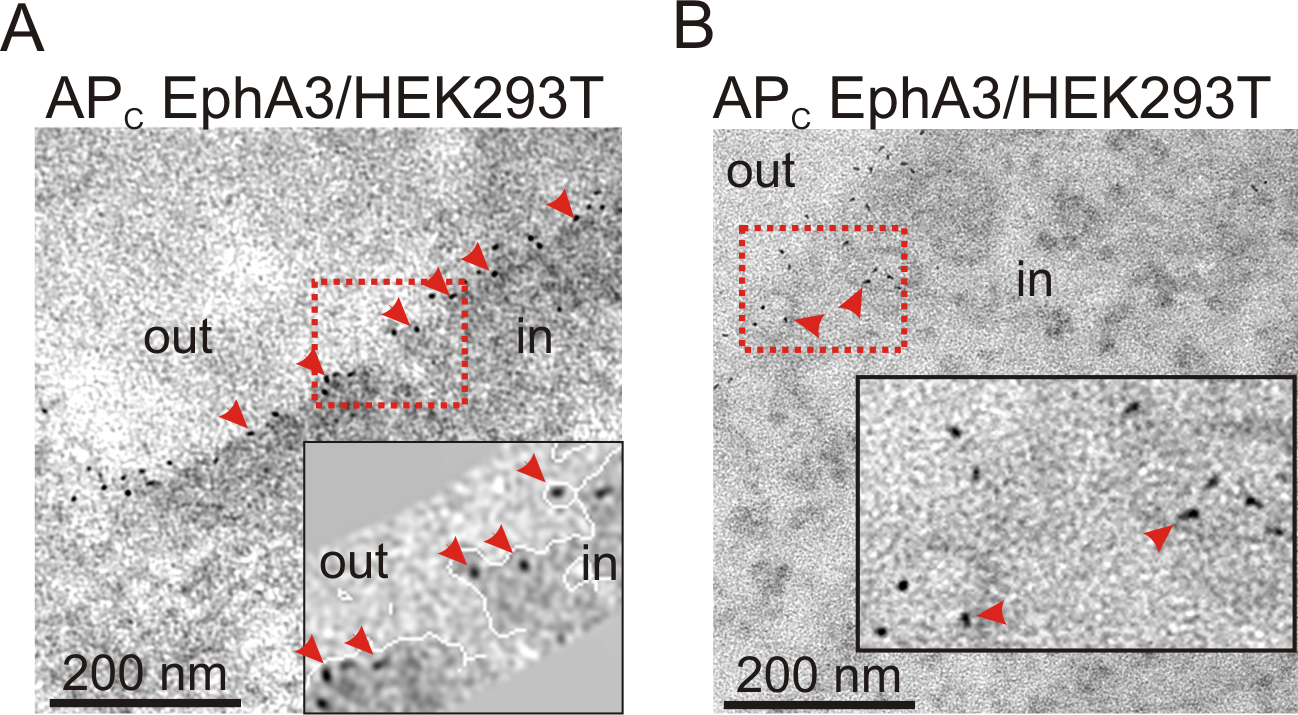

Supplement: Figure S10 — EM of Qdot-labelled EphA3. EM images of fixed APC-EphA3-expressing HEK293T cells without pretreatment (A) or following ephrin stimulation (B), showing plasma membrane disruption and endocytosis, respectively. Cells were fixed, permeabilized, and stained with SA-Qdots 605 prior to EM plastic embedding. Inserts show an enlarged section of the boxed (red) areas, using computer-assisted assignment to delineate the exact plasma membrane/cytoplasm boundary. Arrowheads mark EphA3-tethered Qdots on the inner cell membrane. (1.01 MB TIF) [file pbio.1000215.s010.tif]
